# Immediate Neutrophil-Variable-T Cell Receptor Host Response in Bacterial Meningitis

**DOI:** 10.3389/fneur.2019.00307

**Published:** 2019-04-02

**Authors:** Tina Fuchs, Kerstin Puellmann, David H. Dreyfus, Armin P. Piehler, Björn Reuter, Christopher Schwarzbach, Olaf Willmann, Diego Yepes, Victor Costina, Peter Findeisen, Jens Mahrt, Chunlin Wang, Jian Han, Alexander W. Beham, Michael Neumaier, Wolfgang E. Kaminski

**Affiliations:** ^1^Institute for Clinical Chemistry, Medical Faculty Mannheim, University of Heidelberg, Mannheim, Germany; ^2^Aesculabor Hamburg, Hamburg, Germany; ^3^Department of Pediatrics, Yale School of Medicine, New Haven, CT, United States; ^4^Bioscientia Institute for Medical Diagnostics, Karlsfeld, Germany; ^5^Department of Neurology, Medical Faculty Mannheim, University of Heidelberg, Mannheim, Germany; ^6^Molecular & Optical Live Cell Imaging, University of Göttingen, Göttingen, Germany; ^7^iRepertoire Inc., Huntsville, AL, United States; ^8^HudsonAlpha Institute for Biotechnology, Huntsville, AL, United States; ^9^Department of Surgery, Hospital of Siegen, Siegen, Germany; ^10^Bioscientia Institute for Medical Diagnostics, Ingelheim, Germany

**Keywords:** bacterial meningitis, T cell receptors, neutrophil granulocytes, V(D)J receptors, phagocytosis

## Abstract

Bacterial meningitis is a life-threatening disease that evokes an intense neutrophil-dominated host response to microbes invading the subarachnoid space. Recent evidence indicates the existence of combinatorial V(D)J immune receptors in neutrophils that are based on the T cell receptor (TCR). Here, we investigated expression of the novel neutrophil TCRαβ-based V(D)J receptors in cerebrospinal fluid (CSF) from human patients with acute-phase bacterial meningitis using immunocytochemical, genetic immunoprofiling, cell biological, and mass spectrometric techniques. We find that the human neutrophil combinatorial V(D)J receptors are rapidly induced in CSF neutrophils during the first hours of bacterial meningitis. Immune receptor repertoire diversity is consistently increased in CSF neutrophils relative to circulating neutrophils and phagocytosis of baits directed to the variable immunoreceptor is enhanced in CSF neutrophils during acute-phase meningitis. Our results reveal that a flexible immune response involving neutrophil V(D)J receptors which enhance phagocytosis is immediately initiated at the site of acute bacterial infection.

## Introduction

Bacterial meningitis is associated with considerable mortality and morbidity worldwide and survivors are at increased risk of experiencing serious long term disabling sequelae (1–5). The pathogenetic mechanisms of this life-threatening infection are complex and still incompletely understood (6–8). A key pathogenetic event in the disease is the massive influx of neutrophils into the subarachnoid space in response to bacterial invasion (1).

We have recently identified an as yet unrecognized recombinatorial immune receptor in human and murine neutrophil granulocytes whose genetic structure is based on the αβ T-cell receptor (9, 10), here referred to as *neutrophil* TCRαβ. The TCRαβ immunoreceptor in neutrophils is expressed across the entire human life span, undergoes a dramatic decline during aging (11) and is implicated in autoimmune disease (12) and malaria infection (13). Further recent studies demonstrate TCR gene rearrangement in mouse granulocytes (14), TCR expression by oral neutrophils in chronic periodontitis (15), and expression of a TCRγδ immunoreceptor by human eosinophils (16) and thus confirm the existence of variable immune receptors in the granulocyte lineage. Consistent with myeloid TCR expression, two recent studies provide compelling evidence that subpopulations of monocytes and macrophages, the second major phagocyte population, also express variable immune receptors that are structurally based on the TCRαβ (17) and the TCRγδ (18), respectively. Separate from this, the variable macrophage TCRαβ has been implicated in atherosclerosis (19), malaria infection (20) and cancer (21). Based on these findings we have put forward the concept of extralymphocytic flexible immune recognition (22, 23). Of note, most recent studies from our laboratory and others demonstrated the expression of the second variable immune receptor based on immunoglobulin heavy and light chain genes by cells of the myeloid lineage (24–26). The discovery of recombined TCR- and immunoglobulin based immune receptors in human and mouse granulocytes and macrophages extends the principle of combinatorial immune recognition to phagocytic cells and strongly suggests that flexible host defense in higher vertebrates may operate on a broader cellular basis than currently thought.

The function of the neutrophil TCRαβ and its involvement in inflammatory disease is ill defined. Here, we tested whether this combinatorial myeloid immune receptor is implicated in acute bacterial meningitis in adults. This infectious disease is a particularly attractive candidate for exploring the role of the neutrophil TCRαβ in clinical pathologies owing to the intense neutrophil response that drives the inflammation and the fact that neutrophils are recruited to a preformed space that is filled with liquid, the cerebrospinal fluid (CSF). Unlike other sites of purulent infection, the latter provides conditions in which degraded tissue is absent and thus facilitates precise analysis of the immune function of neutrophils at the site of inflammation.

## Materials and Methods

### Patients, Probands, and Sample Collection

Adult patients were recruited to the study in the random order they were admitted to the emergency rooms or the intensive care units of the Hospital of the University of Heidelberg, Campus Mannheim. Inclusion criteria were: (i) suspected bacterial meningitis, (ii) initial CSF leukocyte count ≥300/μl, (iii) CSF neutrophil fraction >75%, (iv) age >17 years, and (v) positive CSF cultures for bacterial pathogens. Under these conditions, a total of 10 patients (5 females, 5 males, Table S1) were recruited within a period of 9 months who met *all* of the above criteria. In addition, one patient meeting the above criteria was included for neutrophil activation experiments, and one patient with *varicella zoster* meningitis was recruited as a positive control representing an acute non-bacterial meningitis. All patients were free of immunosuppressive medication. One patient (patient 5) was diagnosed with monoclonal gammopathy shortly after admission to the hospital. A total of 10 healthy individuals was randomly selected for additional neutrophil experiments.

The samples analyzed included acute-phase CSF obtained by lumbar puncture and peripheral blood collected by venipuncture at the same time point that were submitted to the laboratory for routine testing. From residual aliquots of CSF and peripheral blood CD15^+^ neutrophil and CD3^+^ T cell were isolated for further analysis. In two patients with pneumococcal and meningococcal meningitis (patient 4 and 5) CSF and peripheral blood samples were collected at a second time point 5 and 7 days after symptom onset, respectively, to monitor the response to antimicrobial therapy. All CSF and peripheral blood samples were processed within 3 h of collection. The study was approved by the Ethics Committee of the Medical Faculty Mannheim, University of Heidelberg (Permit Number: 2007-254N-MA). Written informed consent had been obtained from all individuals included in this study.

### Pathogen Identification

Bacterial pathogens in the CSF samples were identified by light microscopy of Wright-Giemsa or Gram stained cytospin smears, microbiological culturing techniques, and MALDI-TOF biotyping, respectively. Viral pathogens were detected using routine PCR testing.

### Cell Purification and Flow Cytometry

CD15^+^ neutrophils and CD3^+^ lymphocytes were purified by density gradient centrifugation (Ficoll-Paque, GE Healthcare) and magnetic cell sorting (MACS, Miltenyi Biotec) from freshly obtained CSF and peripheral blood that was drawn at the same timepoint. Purity of CD15-MACS isolated neutrophils was routinely >99.5% as assessed by flow cytometry (representatively shown in **Figures 2B**, **3**). Light microscopic inspection of Giemsa stained CD15^+^ cytospin preparations from all patients by two independent trained hematologists (KP, WEK) confirmed these results. Viability of CD15-MACS purified neutrophils was >98% for up to 12 h even in the presence of bacterial pathogens as assessed by flow cytometric 7-AAD staining (data not shown). Flow cytometric analyses were performed on a FACSCalibur flow cytometer (BD Biosciences) using the following antibodies: anti-human CD3-APC, anti-human CD15-PE, anti-human CD2-FITC, and anti-human CD19-PercP (BD Biosciences).

### Immunocytochemistry and Confocal Microscopy

For immunostaining, cytospins were fixed for 10 min in icecold acetone. After 20 min of drying the cells were blocked with 5% goat serum in 1% BSA in PBS, incubated with mouse anti-human monoclonal anti-TCRβF1 (clone 8A3; 1:50, Thermo Scientific), rabbit anti-human CD11a, rabbit anti-human CD11c, rabbit anti-human CD18, rabbit anti-human CD83 (all 1:100; antibodies-online) or mouse anti-human CD11b (1:100; BD Biosciences) at 4°C overnight, washed in PBS for 15 min, and incubated with Alexa 488-labeled goat anti-mouse IgG (1:200, Invitrogen), Cy3-labeled donkey anti-rabbit IgG (1:200, Jackson Immunoresearch), and DRAQ5 (1:2000, Alexis), respectively. Mouse IgG1 (BD Biosciences) or rabbit IgG-isotype control antibodies (Abcam) were used as negative control. Positive staining was visualized by confocal microscopy as previously described (17). For 3D analyses, microscopic confocal imaging was performed using an Olympus FV-1000 5 channel system (System Version 3.1.1.9), equipped with 405 nm, a multiline argon laser, a 561 nm diode, a 594 nm HeNe-laser and a HeNe 633-nm laser, respectively. For 3D volume rendering xy-images (640 × 640 pix, 12 μs/pix) were recorded along the z-axis at 59 nm intervals throughout the entire tissue depth. Images were recorded using a UPLSAPO−60x/1.35 NA oil immersion objective. The respective xyz-stacks of the raw images (Olympus OIB-format) were transformed into 3D volume-rendered data for all fluorescent channels (Alexa 405, Alexa 488 and Alexa 633) using Imaris x64, version 7.4.0 (Bitplane) and exported as 3D rotation movies.

### Immunoblotting and Mass Spectrometry

SDS-PAGE and transfer to nitrocellulose membranes was conducted as previously reported (9, 11, 17). For detection of the TCRαβ the mouse anti-human TCRβF1 framework antibody was used (Thermo Scientific) in combination with a HRP-conjugated rabbit anti-mouse IgG (Invitrogen). A rabbit polyclonal antibody to β-actin was used for detection of a housekeeping gene loading control (Abcam). Visual quantification of the TCRβ bands in relation to the actin signals were performed by using the Image Lab™ software (BioRad). MALDI-TOF mass spectrometry was performed on immunoprecipitated TCRαβ samples which were separated by SDS-PAGE. Sample preparation, measurement and analysis was performed as previously described (17).

### IL-8 ELISA

Interleukin-8 levels were measured in serum and CSF samples collected from patients with acute bacterial meningitis and healthy age-matched controls using a Human IL-8 Single Analyte ELISArray Kit (SABiosciences) according to the manufacturers' protocol.

### TCR Vβ CDR3 Length Spectratyping and Clonotype Sequencing

In order to assess the TCR Vβ repertoire diversity in CD15^+^ neutrophils and CD3^+^ lymphocytes the “CDR3 length spectratyping” method published by Pannetier et al. (27) was used. For this, the CDR3_β_ regions of all known Vβ chains (*n* = 25) were PCR amplified and subsequently fluorescence labeled in a run-off reaction for 4 cycles using the D4-labeled primer D4-TTGGGTGTGGGAGATCTCTGC specific for the TCRβ constant region. The expressed Vβ length variants (“spectratypes”) were analyzed on a CEQ™ 8000 Genetic Analysis System (Beckman Coulter) (9, 11, 17). To determine the detailed CDR3 clonotype sequences for selected Vβ chains expressed by PB- and CSF neutrophils, specific RT-PCR amplification products were cloned into a pCR-TOPO vector (TOPO TA Cloning Kit, Invitrogen) and the CDR3_β_ sequences were analyzed from at least 10 randomly picked clones as previously described (17).

### TCRβ Immune Repertoire Deep Sequencing

Total RNA of the PB and CSF cells was extracted with Trizol reagent and subjected to RT-PCR using a One-step RT-PCR kit (Qiagen). cDNA was then subjected to Amplicon rescued multiplex PCR (ARM-PCR) using human T cell beta receptor primers according to the manufacturer's instructions (iRepertoire Inc.). The PCR products were run on 2% agarose gel and the DNA was extracted from the gel using a Gel Extraction kit (Qiagen). After library preparation high-throughput sequencing of the CDR3 regions was performed on a HiSeq2000 DNA sequencer (Illumina) by iRepertoire Inc. The raw data were analyzed by iRepertoire using IRmap programs with a modified Smith-Waterman algorithm. For data analyses, paired-end sequence reads were joined together and the merged sequences were mapped to germline V, D and J reference sequences according to the method we previously reported (28). A newly developed five-step SMART filtering strategy was applied to the NGS data (Wang et al., see accompanying manuscript). This advanced computational approach utilizes a cascade of filtering algorithms that eliminate artifactual sequences at five distinct quality control checkpoints. The SMART strategy, which can detect >99% of erroneous sequences in the immune repertoire, is composed of the following five filters: (i) sequencing error filter, (ii) mosaic sequencing filter, (iii) PCR amplification performance filter, (iv) reference sequence filter, and (v) frequency threshold filter, respectively. The SMART strategy was employed on all mapped CDR3 reads and the frequency threshold filter was set to >1. Consequently, all single copy TCRβ CDR3 sequence variants were discarded.

### Infection of Neutrophils With Bacterial Pathogens

Bacteria [*S. pneumoniae* (ATCC 47619), *N. meningitidis* (group B, wildtype)] were propagated overnight, resuspended in RPMI 1640 (PAA Laboratories) and subsequently heat-inactivated for 1 h at 65°C. Freshly isolated CD15^+^ neutrophils from three healthy individuals (two females 28y/63y, one male 47y) were then infected with the bacteria at a ratio of 1:10. After 9 h incubation at RT cells were harvested in TRI Reagent BD (Sigma-Aldrich).

### TCRαβ-Directed Phagocytosis Assay

Aliquots of 3 x 10^6^ freshly collected CD15^+^ PB neutrophils or CSF neutrophils were incubated in the presence of standardized polystyrene bead baits (Ø 4.5 μm, Invitrogen) for 1.5–3 h at room temperature in RPMI medium (cell/bead ratio 1:1). Bead baits were coated with endotoxin-free anti-TCRαβ antibodies (5 μg per 10^7^ bead baits) or equal quantities of endotoxin-free non-specific IgG antibodies (isotype control) and albumin (irrelevant protein), respectively. Subsequently, cytospins were prepared and neutrophil bead phagocytosis was quantitated by bright field microscopy. For blockade of phagocytosis cytochalasin D was used at a concentration of 5 μg/ml. Neutrophils that had ingested or bound ≥1 bead were considered phagocytosis positive. The total number of beads associated with the cell (bound and ingested) was determined for each individual cell. For each cytospin preparation the beads/cell ratio and the percentage of phagocytosing cells were assessed from at least 12 randomly selected fields of vision.

### Phagocytosis and Respiratory Burst Assays

For quantitative determination of neutrophil phagocytic and respiratory burst activities modified protocols from the flow cytometry-based Phagoburst kit (Glycotope Biotechnology) were used. To assess phagocytic activity, aliquots of 10^6^ purified PB neutrophils from healthy individuals (*n* = 5) were pre-incubated with anti-CD3ε (10 μg/ml, clone: OKT3, eBioscience) and anti-CD28 (10 μg/ml, clone: L293, BD Biosciences) antibodies or non-specific IgG antibodies (isotype controls) for 10 min, and subsequently infected with FITC-labeled non-opsonized *E. coli* bacteria (ATCC25922, cell/bacteria ratio 1:10) for 10 min. Respiratory burst activity was assessed in the presence of PMA (1 μg/ml), CD3/CD28 costimulation, non-specific IgG or the absence of a stimulus. All incubations were performed in serum-free medium at 37° C.

## Results

**Figure 1:**
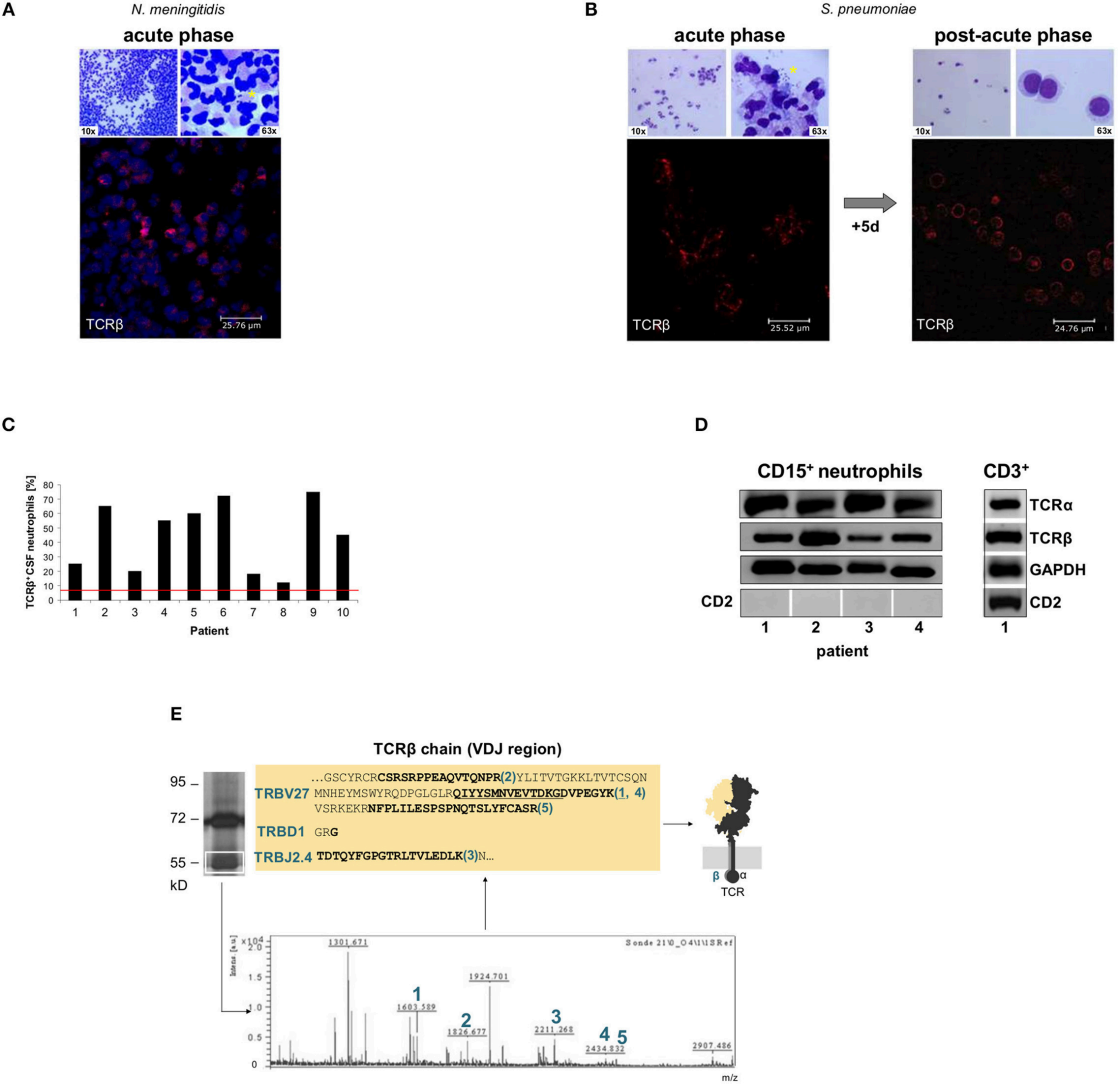
Rapid accumulation of T cell receptor αβ bearing neutrophils (TCRαβ^+^) in the cerebrospinal fluid (CSF) of patients with acute bacterial meningitis. CSF cytology images from two representative patients with acute bacterial meningitis caused by *Neisseria meningitidis* **(A**, patient 4**)** and *Streptococcus pneumoniae*, respectively **(B**, patient 5**)**. The time between onset of specific symptoms and CSF collection was 9–15 h. The top row in each panel represents Giemsa stained CSF-cytospin preparations that demonstrate the massive accumulation of neutrophils (left). The presence of bacteria (asterisks) is shown at a higher magnification (right). Confocal immunofluorescence microscopy of CD15^+^-MACS purified CSF neutrophils (bottom) reveals consistent accumulation of TCRαβ expressing neutrophils (red) during the acute phase of the inflammatory response. Nuclei in panel A (blue) are counterstained with DRAQ5. CSF cytology in **(B)** is also shown after 5 days of antibiotic therapy revealing the presence of the TCRαβ in the neutrophil-driven acute phase and TCRαβ expression by T cells in the lymphocyte dominated post-acute phase. See also Figures S1, S2, S4 for CSF cytology images of additional patients. Isotype controls are shown in Figure S3. **(C)** Percentages of CSF neutrophils that stained positive for the TCRαβ in a cohort of 10 randomly selected patients with bacterial meningitis. Quantitative analysis of TCRαβ staining was performed in CD15^+^-purified CSF neutrophils that were collected during acute-phase bacterial meningitis. The red line represents the average percentage of TCRαβ^+^ neutrophils present in the circulation of healthy individuals. **(D)** RT-PCR demonstrates expression of the TCRα and the TCRβ constant chain genes in CSF CD15^+^-neutrophils from patients with acute-phase bacterial meningitis. Four patients (1–4) are representatively shown. CSF T cells (CD3^+^) from patient 1 are shown as positive control. Absence of expression of the lymphoid marker CD2 demonstrates that CD15^+^-MACS purified neutrophils were free of NK or T cells. GAPDH, loading control. **(E)** Direct mass-spectrometric identification of a rearranged Vβ-chain variant in CSF neutrophils from patient 2 (*S. pneumoniae* meningitis). Protein lysates from the patient's CSF CD15^+^-neutrophils were immunoprecipitated using an anti-TCRβ antibody and the predicted 58 kD band (boxed) was analyzed by MALDI-TOF mass spectrometry. Peaks 1–5 represent TCR Vβ-specific peptide fragments whose amino acid sequence identities with known TCR Vβ-chains are bolded. They are consistent with a TRBV27-TRBD1-TRBJ2.4 rearranged clonotype. For protein identification, peptide mass fingerprints were searched in the MASCOT database. See also Figure S7.

Leukocyte counts in the 10 CSF samples recruited to the study ranged from 300 to 15,418 cells/μl (Table S1). The time between onset of specific symptoms and CSF collection was 9–15 h. Light microscopy of Giemsa stained CSF cytospin preparations showed the characteristic cytological picture of acute bacterial meningitis with massive presence of neutrophils (>80%) and near absence of lymphocytes in all specimens (Figures 1A,B, S1). This contrasts with viral meningitis which is typically dominated by lymphocytes known to express the TCR (Figure S2). Bacteria were detectable by light microscopy in all cases with the exception of patient 1. Culturing provided evidence for the presence of bacterial pathogens in the CSF of all patients (Table S1).

Light microscopic inspection confirmed that all CD15-purified and the vast majority of the unpurified CSF-cells displayed the typical morphological appearance of human neutrophils. No morphological alterations were observed that would suggest “transdifferentiation” of neutrophils (Figures 1, S1, S3–S5). To further substantiate that CD15^+^ CSF-leukocytes were authentic neutrophils, we tested for surface expression of additional neutrophil markers. Because PB neutrophils have been shown to adopt characteristics of dendritic cells (DC) under specific long-term culturing conditions (29), we also investigated expression of the DC marker CD83. Immunocytochemistry revealed constitutive expression of the canonical neutrophil markers CD11a, CD11b, CD11c and CD18, respectively, in the patients' CD15^+^ CSF neutrophils but not expression of CD83 (Figure S6A). Consistent with this, exposure to septic CSF for 14 h freshly collected from an additional patient did not alter neutrophil-specific lineage marker expression in PB neutrophils (Figure S6B). These findings indicate that neutrophils retain their typical lineage-specific marker profile in the subarachnoid space during acute bacterial meningitis.

Immunostaining demonstrated the presence of TCRαβ bearing neutrophils in the CD15^+^ CSF cytospins of all 10 patients tested (Figures 1A,B, S1, S3). In the post-acute-phase, 5–7 days after the initial puncture and antibacterial therapy, neutrophils were replaced by TCRαβ bearing T-lymphocytes (Figures 1B, S4). Quantitative analysis of the confocal immunofluorescence images revealed that 12–75% of the CSF neutrophils displayed TCRαβ staining (Figure 1C) indicating a 2–15 fold increase in TCRαβ expressing neutrophils in the CSF compared to PB neutrophils. In the latter, the TCRαβ^+^ fraction ranged from 5 to 8% (Figure S5) similarly as previously observed in normal individuals (9).

In keeping with immunocytochemistry, RT-PCR demonstrated expression of both the TCRα and -β constant chain genes in the acute-phase CSF neutrophils (Figure 1D). Immunoprecipitation of the TCR from CD15^+^ purified CSF neutrophils of three patients followed by MALDI-TOF mass spectrometry identified peptides that showed partial sequence identity with known variable TCR β-chain fragments (Figures 1E, S7) thus providing independent protein profiling evidence for TCR expression in CSF neutrophils. Of note, all of the peptides (TRBV27–TRBD1–TRBJ2.4, TRBV12-5–TRBD1, TRBV–TRBD1–TRBJ2.7–C) spanned V → J and/ or J → C junctions indicating that they originated from rearranged TCRβ gene loci. Consistent with this, genomic analyses routinely demonstrated V(D)J rearrangements in the TCRβ gene locus of human neutrophils (Figure S8).

The combined data from immunocytochemistry, gene expression profiling and mass-spectrometry protein profiling thus establish that a large subpopulation of neutrophils that invade the subarachnoid space during acute bacterial meningitis express a variable TCRαβ immune receptor.

**Figure 2:**
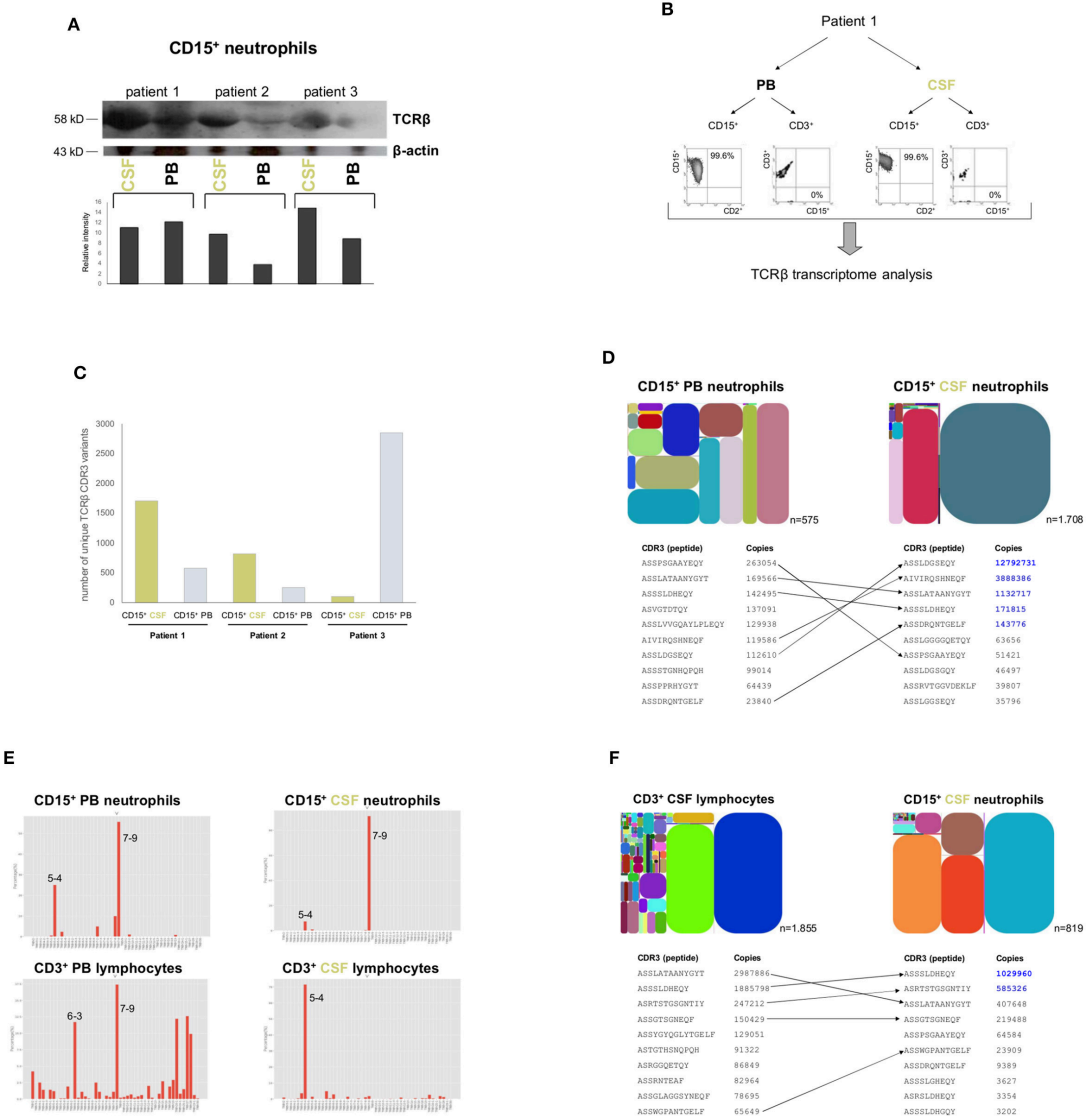
Induction of the variable TCRαβ in CSF neutrophils during the acute phase of bacterial meningitis**. (A)** The Western blot shows TCRαβ levels in CD15^+^ neutrophils from acute phase CSF (<15 h post-onset of symptoms) and simultaneously collected peripheral blood (PB) in patients with bacterial meningitis caused by *S. aureus* (patient 1), *S. pneumoniae* (patient 2) and *E. coli*, respectively (patient 3). A quantitative analysis of the TCRβ western blot signals in relation to β-actin are shown below. β-actin, protein loading control. The original blots this figure is based on are displayed in the Supplementary Material. **(B)** Experimental design of dual lineage NGS-based TCRβ transcriptome analyses in patients with acute bacterial meningitis (representatively shown for patient 1). Highly pure CD15^+^ neutrophils and CD3^+^ lymphocytes were obtained simultaneously from the patients' peripheral blood and CSF, respectively, and TCRβ transcriptomes were analyzed by ARM-PCR based high-throughput sequencing. The scattergrams document the purity of the isolated cell fractions. **(C)** Sequencing of the TCRβ transcriptomes of patients 1–3 reveals that the unique number of expressed TCRβ CDR3 variants is higher in CD15^+^ CSF neutrophils of patient 1 and 2 than in CD15^+^ PB neutrophils indicative of repertoire broadening at the site of inflammation. Shown are the unique TCRβ CDR3 variants. A unique CDR3 sequence is defined as a non-redundant fragment of amino acids which is derived from a stop-codon-free reading frame containing both translated conserved V and J motifs. An overview of the NGS results including effective reads, unique and total CDR3 numbers are shown in Table S2. **(D)** (top) Repertoire diversity tree plots visualize the relative abundance of the TCRβ CDR3 transcript variants that are expressed by CD15^+^ neutrophils in CSF and in peripheral blood (patient 1). Each spot represents a rearranged TCRβ transcript that encodes a unique CDR3_β_ sequence. It is defined by a unique color and its area is proportional to the relative transcript frequency. The position of each spot within the plot area is defined according to its Vβ usage (x-axis: Vβ_1_ → Vβ_i_) and Jβ usage (y-axis: Jβ_1_ → Jβ_i_). Each plot has a distinct color code. Total numbers of identified non-redundant (“unique”) TCRβ CDR3 sequence variants are indicated for each diversity tree plot. CDR3, complementarity determining region 3. (bottom) Detailed list of the 10 most frequently expressed TCRβ CDR3 variants in each neutrophil population. Transcript copy numbers are indicated. TCRβ variants that are shared between PB and CSF neutrophils are connected by arrows. TCRβ variants that show an increase in CSF neutrophils are highlighted in blue. See also Figure S13. **(E)** Vβ gene usage of CD15^+^ neutrophils and CD3^+^ lymphocytes, respectively, in peripheral blood and CSF (patient 1). The 2D-plots demonstrate the relative usage of the Vβ genes for each cell compartment. This analysis is based on the number directly observed from the read count data. Note the markedly restricted Vβ gene usage in CSF cells relative to PB cells. X-axis: Vβ gene; y-axis: percentage of used Vβ genes. A normalized distribution of the Vβ gene usage is shown in Figure S15. **(F)** (top) Relative abundance of TCRβ CDR3 transcript variants expressed by CD15^+^ neutrophils and CD3^+^ lymphocytes in CSF (patient 2). Total numbers of identified non-redundant TCRβ CDR3 sequence variants are indicated for each leukocyte subpopulation. (bottom) Comparison of the 10 most frequently expressed TCRβ CDR3 variants reveals that neutrophils and lymphocytes in CSF share common repertoires (arrows), but also express a similar proportion of distinct TCRβ variants. Numbers designate transcript copy numbers. Note that two of the shared TCRβ CDR3 sequence variants exhibit higher expression rates in CSF neutrophils than in CSF lymphocytes (blue). See also Figure S16.

We next tested whether and to which degree neutrophil TCRαβ expression in CSF represents a dynamic local response to bacterial infection or merely reflects the status of the neutrophil host defense that currently prevails in the circulation. For this, we compared TCRαβ expression levels between CSF neutrophils and PB neutrophils that were simultaneously collected from three patients with meningitis (caused by *S. aureus, S. pneumoniae*, and *E. coli*, respectively). Importantly, Western blot demonstrated that TCRαβ protein levels were markedly increased in the CSF neutrophils compared to PB neutrophils irrespective of the causative bacterial pathogen indicating that the TCRαβ is induced in the CSF during acute bacterial meningitis (Figure 2A). Consistent with their high activation state, levels of the major neutrophil chemoattractant IL-8, representatively assessed in three patients (8–10), were dramatically increased in the CSF (Figure S9). Global assessment of the combinatorial diversity of the TCR Vβ chain by using deep sequencing based TCRβ transcriptome analysis confirmed that the total numbers of expressed CDR3_β_ variants were markedly higher in CSF neutrophils from patient 1 and 2 compared to PB neutrophils. Of note, the divergent numbers of unique and total CDR3_β_ variants obtained by deep sequencing were established by using a semi-quantitative approach in combination with stringent quality filtering to eliminate PCR and sequencing artifacts. As a consequence, the results indicate that in these meningitis patients repertoire broadening had occurred in CSF neutrophils (Figures 2B,C and Table S2). Conventional CDR3 spectratyping analysis and Sanger sequencing confirmed these findings (Figures S10–S12). Detailed comparison of the 10 most highly expressed CDR3_β_ variants between CSF neutrophils and PB neutrophils demonstrated that in each compartment neutrophils expressed common but also distinct TCRβ variants. Importantly, the commonly expressed variants displayed a marked induction in CSF (Figures 2D, S13, S14). Analysis of the Vβ gene usage in neutrophils from CSF and PB and T cells from the same compartments showed a clearly restricted Vβ gene usage in CSF cells compared to PB cells (Figures 2E, S15).

Similarly as observed for CSF neutrophils and PB neutrophils, comparison of the 10 most highly expressed CDR3_β_ variants between CSF T cells and neutrophils in two patients revealed expression of both common but also distinct repertoires. This indicates that the acute flexible immune response mounted in CSF is cell-type specific (Figures 2F, S16).

Collectively, these results reveal that the recombinatorial neutrophil TCR immune receptor is markedly induced in the CSF during the first hours of bacterial meningitis.

**Figure 3:**
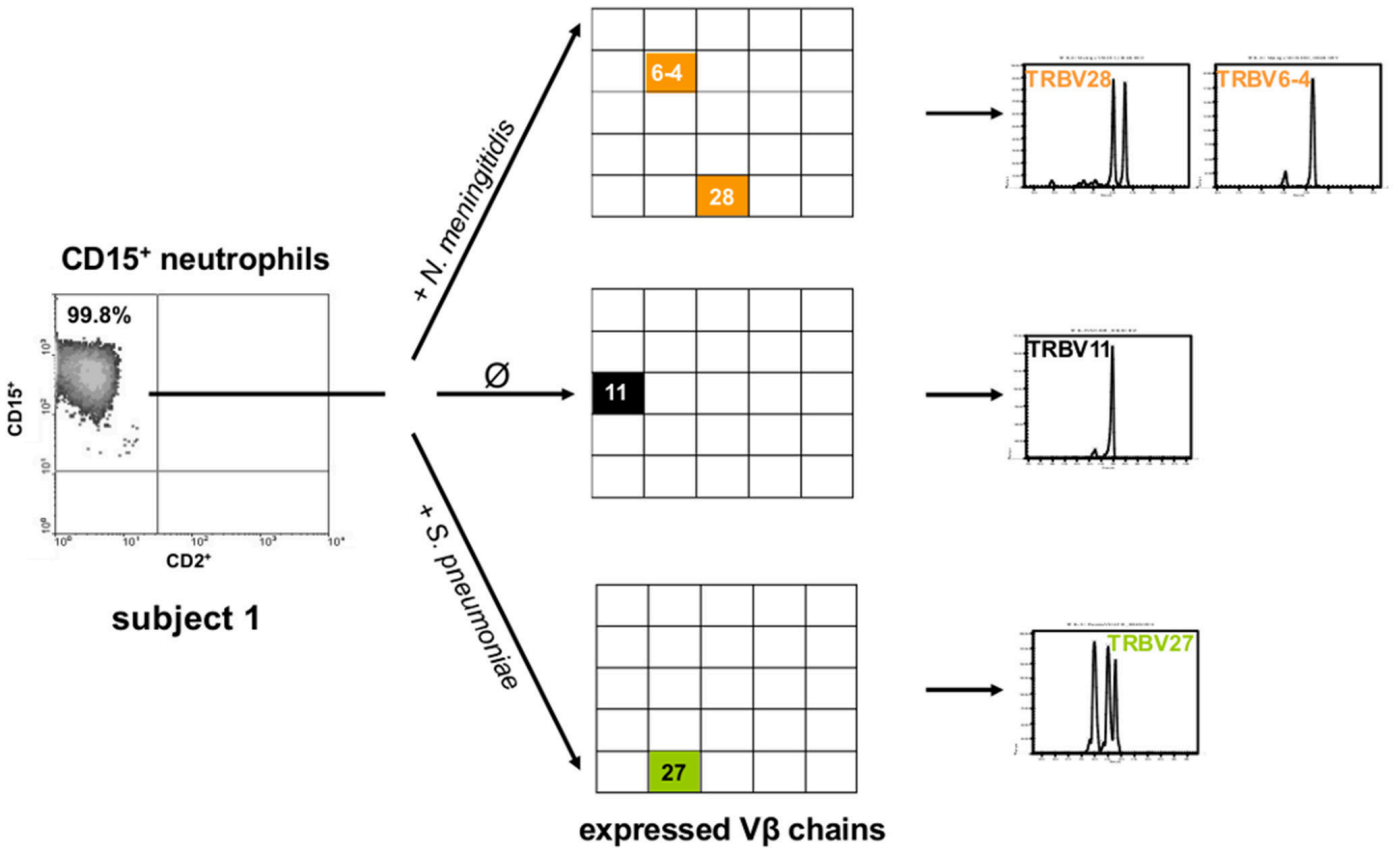
Exposure of neutrophils to bacterial pathogens that cause meningitis induces TCR Vβ repertoire changes *in vitro*. Shown are the TCR Vβ repertoires that are expressed by peripheral blood CD15^+^ neutrophils from a healthy subject after 9 h incubation with *N. meningitidis* (orange), *S. pneumoniae* (green) and in the absence of a bacterial pathogen, respectively (Ø, control). CD15^+^ neutrophils were obtained from a single blood draw. The detailed CDR3_β_ length variants are shown in the right panel. The scattergrams (left) document the purity of the CD15^+^ neutrophils. Note that *N. meningitidis* and *S. pneumoniae* induce distinct TCR Vβ chain usage (center) and CDR3_β_ repertoires (right). See also Figure S17.

To explore the dynamics of the rapid TCR induction in CSF neutrophils, we tested *ex vivo* whether bacteria that cause meningitis are capable of modulating TCR repertoire expression. In fact, we found that exposure of PB neutrophils from healthy subjects to *N. meningitidis* or *S. pneumoniae* for only 9 h was sufficient to induce changes in the expressed neutrophil TCR repertoire patterns (Figures 3, S17).

**Figure 4:**
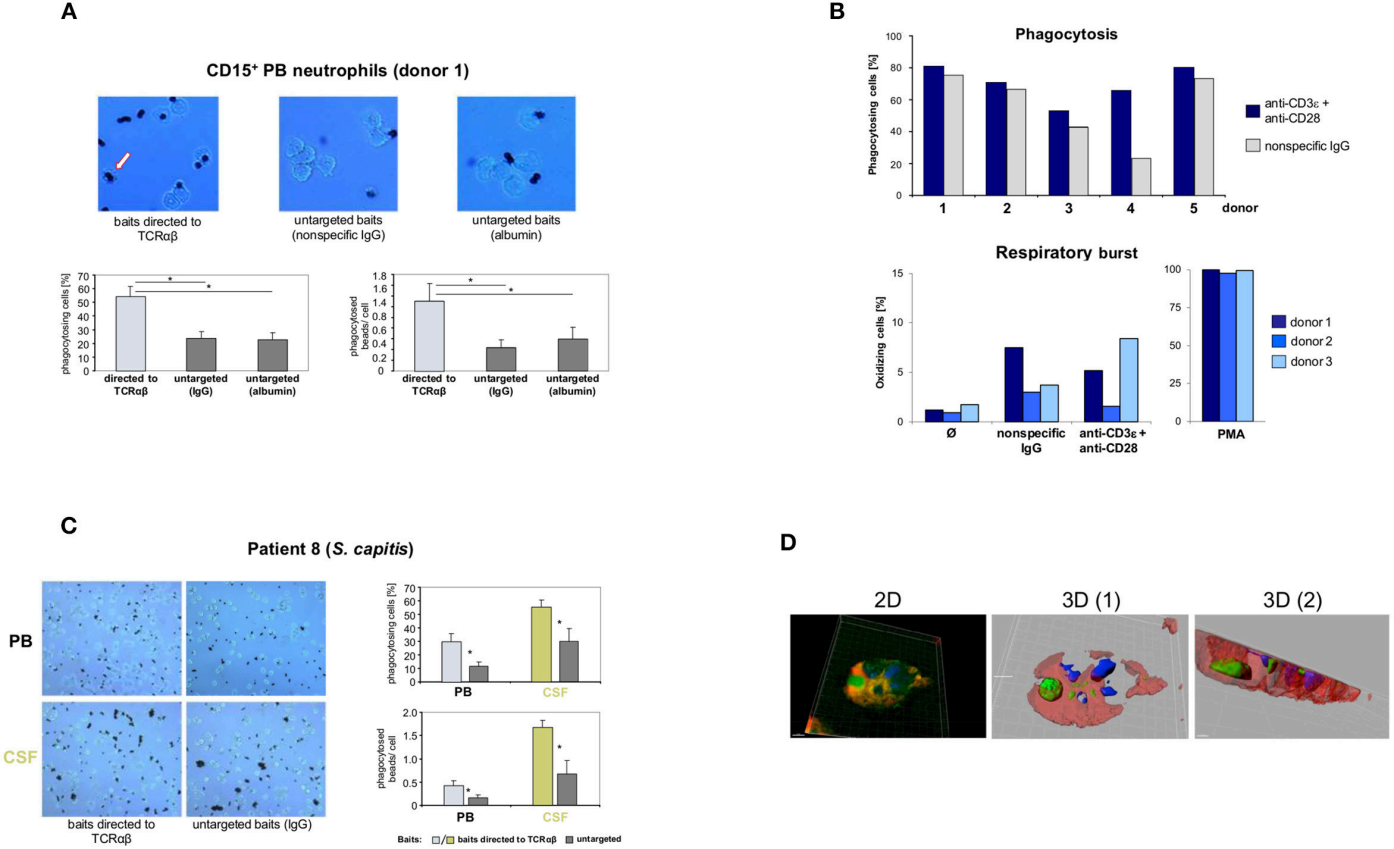
Targeting of baits to the TCRαβ and TCRαβ activation via CD3/CD28 costimulation enhance neutrophil phagocytosis. **(A)** Representative unstained cytospin preparations (40x) of PB neutrophils from a healthy subject (donor 1) that were challenged with bead baits targeted to the TCRαβ or untargeted beads (controls) for 3 h. The arrow (left) highlights two phagocytosed beads. The quantitative analysis of neutrophil bead phagocytosis, defined as ingestion or binding of ≥1 bead, is shown in the bottom panel (percentage of phagocytosing cells, left; phagocytosed beads/cell, right). Note that targeting of beads to the TCRαβ significantly enhances PB neutrophil phagocytosis relative to untargeted beads. Purified PB CD15^+^ neutrophils were incubated with polystyrene bead baits (Ø 4.5 μm) coated with anti-TCRαβ antibodies and uptake of beads was recorded. Beads coated with equal amounts of non-specific IgG isotype antibodies, potentially binding to the F_c_γ receptor, or albumin (irrelevant protein) served as controls (untargeted baits). See also Figures S18, S19 in the Supplementary material. **(B)** Anti-CD3/CD28-mediated TCRαβ activation enhances F_c_ receptor-independent phagocytosis of bacteria (top) but not respiratory burst in neutrophils (bottom). PB CD15^+^ neutrophils from five healthy individuals were infected for 10 min with non-opsonized FITC-labeled *E. coli* baits (cell/bacteria ratio 1:10) in the presence of anti-CD3ε/CD28 antibodies or non-specific IgG (top). Shown are the percentages of phagocytosing neutrophils. The bottom panel shows that CD3/CD28 costimulation under non-opsonizing conditions (three donors) has no effect on the neutrophil respiratory burst. Phagocytic and respiratory burst activities were assessed by flow cytometry. PMA, phorbol myristate acetate. **(C)** Increased phagocytosis of baits targeted to the TCRαβ in PB neutrophils and CSF neutrophils from a patient with *Staphylococcus capitis* meningitis (patient 8). PB and CSF neutrophils were simultaneously collected from the patient during the early phase of meningitis and subsequently challenged with beads targeted to the TCRαβ or untargeted beads (non-specific IgG) for 1.5 h. Representative cytospin preparations (20x, left) and the quantitative analysis of bead phagocytosis are shown (right). **(D)** 3D confocal immunofluorescence imaging identifies a CSF neutrophil from the same patient that has ingested a bead bait (green) targeted to the TCR. Two distinct cross-sections are shown (center, right). The 2D view is shown left. For a full 3D view see Movie S1. The bead bait was stained with FITC-labeled antibodies to mouse IgG, the cell membrane (red) and the nucleus (blue) were stained with Alexa 633-conjugated WGA and DAPI, respectively. All peripheral blood or CSF neutrophils were CD15^+^-MACS purified and incubated with bead baits at a cell density of 3 × 10^6^/ml and a cell/bead ratio of 1:1. Quantitation of phagocytosed beads was conducted by bright field microscopy of at least 12 randomly selected fields of vision. Error bars represent mean ± SD. ^*^p < 0.001, Dunnett *post-hoc* test **(A)**, Student's pair test **(C)**.

Next, we tested the possibility that the neutrophil TCRαβ interferes with the phagocytic activity of neutrophils. For this, freshly purified CD15^+^ neutrophils from two healthy donors were challenged with standardized phagocytosis baits (polystyrene beads, Ø 4.5 μm) for 3 h (Figure S18). To induce interaction of the baits with the TCRαβ, the baits were coated with anti-TCRαβ antibodies. Identical baits coated with equal amounts of non-specific IgG isotype antibodies or albumin served as reference. Using this *ex vivo* approach, we consistently observed a >2-fold increase in the number of phagocytosing neutrophils (donor 1: 53.8 ± SD vs. 23.8 ± SD; donor 2: 42.2 ± SD vs. 20.8 ± SD) and the phagocytosed bead/cell ratios (donor 1: 1.3 ± SD vs. 0.4 ± SD; donor 2: 1.1 ± SD vs. 0.3 ± SD) in both individuals when TCRαβ-directed phagocytosis was induced (Figures 4A, S19). This marked prophagocytic effect was not fully abrogated when phagocytosis was suppressed by cytochalasin D (Figure S19).

To determine whether neutrophil phagocytosis is directly modulated by the TCRαβ, we next tested the effect of the TCR agonists CD3 and CD28 on human neutrophil phagocytic activity. For this, CD15^+^ neutrophils from healthy individuals were incubated for 10 min in the presence of anti-CD3ε/CD28 antibodies or non-specific IgG (isotype controls) and subsequently infected with non-opsonized fluorescence-labeled *E. coli* baits. In addition, we investigated whether CD3/CD28 mediated costimulation of the TCR augments the respiratory burst activity of neutrophils. CD3/CD28 costimulation resulted in a consistent increase (1.1–2.8 fold) in the percentage of phagocytosing neutrophils in all individuals (Figure 4B). In contrast, TCR costimulation had no effect on neutrophil respiratory burst. These results demonstrate that direct stimulation of the TCR/CD3 complex augments the phagocytic activity of neutrophils in an F_c_ receptor-independent fashion.

Finally, we investigated whether the observed increase in TCRαβ-directed phagocytosis in normal neutrophils was also detectable in neutrophils during acute meningitis. For this, circulating neutrophils and CSF neutrophils from an individual with suspected acute bacterial meningitis (patient 8) were collected upon admission and immediately challenged with TCRαβ-directed baits. As expected by the general activation of neutrophils in the subarachnoid space, phagocytic activity was generally increased in CSF neutrophils compared to PB neutrophils (Figures 4C,D). As observed in the normal subjects, both peripheral blood and CSF neutrophils from the patient displayed increased phagocytosis when baits were directed to the neutrophil TCRαβ (% phagocytosing cells: PB: 29.3 ±SD vs. 11.5 ±SD; CSF: 55.2±SD vs. 30.3 ±SD; phagocytosed bead/cell ratios: PB: 0.4 ±SD vs. 0.2 ±SD; CSF: 1.7±SD vs. 0.7 ±SD). Consistent with the elevated TCRαβ^+^ neutrophil counts in the CSF (CSF 12 vs. PB 5%) TCRαβ-directed phagocytosis was significantly increased in CSF neutrophils relative to PB neutrophils. Collectively, these findings demonstrate that phagocytosis is markedly enhanced in normal and CSF neutrophils when baits are directed to the TCRαβ.

## Discussion

We report that the acute phase of bacterial meningitis is characterized by immediate initiation of a flexible host response in the subarachnoid space that relies on the recently identified neutrophil TCRαβ immune receptor. This is the first demonstration that variable neutrophil based host defense mechanisms are implicated in the initial phase of bacterial infection.

In all patients investigated the induction of the variable neutrophil TCR immune receptor occurred within the first hours of infection and involved three components: First, markedly increased numbers of TCRαβ expressing neutrophils in the subarachnoid space compared to neutrophils in the circulation. We find that the fraction of TCRαβ bearing CSF neutrophils ranges from 12 to 75% whereas only a 5–8% fraction of TCRαβ^+^ neutrophils is routinely present in the circulation (9). Second, CSF neutrophils of patients with acute-phase bacterial meningitis have consistently higher TCRαβ protein levels than those found in the circulating pool. Finally, we noted markedly increased expression of individual TCR Vβ chain repertoires and repertoire broadening in CSF neutrophils relative to circulating neutrophils. This reveals that TCR repertoire changes occur in the CSF neutrophils as early as in the acute phase of bacterial meningitis. The observed effects were not attributable to antimicrobial therapy because in none of the patients treatment was initiated before CSF collection.

Detailed repertoire analyses demonstrate that CSF neutrophils and circulating neutrophils express a fraction of common TCR Vβ variants. These findings suggest that the observed accumulation of large quantities of TCRαβ expressing neutrophils in the CSF during acute-phase bacterial meningitis is the result of recruitment of circulating TCRαβ^+^ neutrophils to the subarachnoid space. However, we routinely found evidence for expression of a significant proportion of additional neutrophil TCR Vβ clonotypes in the CSF that were absent from PB neutrophils indicating that also a rapid and dynamic mobilization of TCR Vβ repertoires occurs in response to bacterial invasion. Consistent with this, we observed that *ex vivo* infection of normal neutrophils with bacteria that cause meningitis triggers changes in TCR repertoire expression within only a few hours.

Canonical CD3/CD28 TCR costimulation resulted in increased phagocytic activity of normal neutrophils. Moreover, we found that targeting of baits to the TCRαβ consistently enhances phagocytosis in normal neutrophils and CSF neutrophils in meningitis. This strongly supports the view that the TCRαβ functions as a facilitator of phagocytosis which is rapidly induced in acute bacterial meningitis. We suggest that induction of a variable neutrophil-based host defense machinery in bacterial meningits is worthy of further study. Given that preliminary results indicate the presence of the TCRαβ in other acute bacterial infections (Fuchs et al., unpublished), we speculate that the findings presented here reflect a general as yet unrecognized host defense mechanism operative in acute bacterial infection.

Conceptually, the observation of an immediate variable TCR response in neutrophils during acute bacterial meningitis identifies a rapidly responding flexible element in the host defense armamentarium of a classic myeloid phagocyte. This lends further evidence to the emerging view that RAG recombinase (30) diversified receptor populations exist outside the lymphocyte lineage in the evolutionarily more ancient population of phagocytes (22, 23). These results are consistent with a more ancient origin of immune recombination since invertebrate organisms such as the sea urchin encode and express functional RAG-1/RAG-2 like proteins that seem to have conserved recombinase function despite the lack of lymphocytes in non-vertebrates. It is thus conceivable that RAG recombination may have preceded the lymphocyte lineage (23, 31, 32).

## Data Availability

The raw data supporting the conclusions of this manuscript will be made available by the authors, without undue reservation, to any qualified researcher.

## Author Contributions

TF, DY, VC, and JM performed the experiments. KP, DD, AP, PF, AB, MN, and WK contributed conception and design of the study. BR, CS, and OW collected clinical data. CW and JH performed next-generation sequencing and bioinformatics. TF, WK, and KP wrote the manuscript and prepared figures and tables. All authors contributed to manuscript revision, read and approved the submitted version.

### Conflict of Interest Statement

The authors declare that the research was conducted in the absence of any commercial or financial relationships that could be construed as a potential conflict of interest.

## References

[1] Kim KS. Pathogenesis of bacterial meningitis: from bacteraemia to neuronal injury. Nat Rev Neurosci. (2003) 4:376–85. 10.1038/nrn1103 12728265

[2] Edmond K Clark A Korczak VS Sanderson C Griffiths UK Rudan I. Global and regional risk of disabling sequelae from bacterial meningitis: a systematic review and meta-analysis. Lancet Infect Dis. (2010) 10:317–28. 10.1016/S1473-3099(10)70048-7 20417414

[3] Stephens DS Greenwood B Brandtzaeg P. Epidemic meningitis, meningococcaemia, and Neisseria meningitidis. Lancet. (2007) 369:2196–210. 10.1016/S0140-6736(07)61016-2 17604802

[4] van de Beek D Drake JM Tunkel AR. Nosocomial bacterial meningitis. N Engl J Med. (2010) 362:146–54. 10.1056/NEJMra0804573 20071704

[5] Oordt-Speets AM Bolijn R van Hoorn RC Bhavsar A Kyaw MH. Global etiology of bacterial meningitis: a systematic review and meta-analysis. PLoS ONE. (2018) 13:e0198772. 10.1371/journal.pone.0198772 29889859 PMC5995389

[6] Brouwer MC de Gans J Heckenberg SG Zwinderman AH van der Poll T van de Beek D. Host genetic susceptibility to pneumococcal and meningococcal disease: a systematic review and meta-analysis. Lancet Infect Dis. (2009) 9:31–44. 10.1016/S1473-3099(08)70261-5 19036641

[7] Coureuil M Mikaty G Miller F Lécuyer H Bernard C Bourdoulous S. Meningococcal type IV pili recruit the polarity complex to cross the brain endothelium. Science. (2009) 325:83–7. 10.1126/science.1173196 19520910 PMC3980637

[8] Rivest S. Regulation of innate immune responses in the brain. Nat Rev Immunol. (2009) 9:429–39. 10.1038/nri2565 19461673

[9] Puellmann K Kaminski WE Vogel M Nebe CT Schroeder J Wolf H From the cover: A variable immunoreceptor in a subpopulation of human neutrophils. Proc Natl Acad Sci USA. (2006) 103:14441–6. 10.1073/pnas.0603406103 16983085 PMC1599981

[10] Puellmann K Beham AW Kaminski WE. Cytokine storm and an anti-CD28 monoclonal antibody. N Engl J Med. (2006) 355:2592–3. 10.1056/NEJMc062750 17167145

[11] Fuchs T Puellmann K Scharfenstein O Eichner R Stobe E Becker A The neutrophil variable TCR-like immune receptor is expressed across the entire human life span but repertoire diversity declines in old age. Biochem Biophys Res Commun. (2012) 419:309–15. 10.1016/j.bbrc.2012.02.017 22342716

[12] Fuchs T Puellmann K Schneider S Kruth J Schulze TJ Neumaier M. An autoimmune double attack. Lancet. (2012) 379:1364. 10.1016/S0140-6736(11)61939-9 22483032

[13] Chorazeczewski JK Aleshnick M Majam V Okoth WA Kurapova R Akue A. TCRβ combinatorial immunoreceptor expression by neutrophils correlates with parasite burden and enhanced phagocytosis during a Plasmodium berghei ANKA malaria infection. Infect Immun. (2018) 86:e00899–17. 10.1128/IAI.00899-17 29685989 PMC6013678

[14] Bell JJ Bhandoola A. The earliest thymic progenitors for T cells possess myeloid lineage potential. Nature. (2008) 452:764–7. 10.1038/nature06840 18401411

[15] Lakschevitz FS Aboodi GM Glogauer M Oral neutrophils display a site-specific phenotype characterized by expression of T-cell receptor. J Periodontol. (2013) 84:1493–503. 10.1902/jop.2012.120477 23205919

[16] Legrand F Driss V Woerly G Loiseau S Hermann E Fournié JJ A functional gammadelta TCR/CD3 complex distinct from gammadelta T cells is expressed by human eosinophils. PLoS ONE. (2009) 4:e5926 10.1371/journal.pone.0005926 19536290 PMC2693924

[17] Beham AW Puellmann K Laird R Fuchs T Streich R Breysach C. A TNF-regulated recombinatorial macrophage immune receptor implicated in granuloma formation in tuberculosis. PloS Pathog. (2011) 7:e1002375. 10.1371/journal.ppat.1002375 22114556 PMC3219713

[18] Fuchs T Puellmann K Hahn M Dollt C Pechlivanidou I Ovsiy I. A second combinatorial immune receptor in monocytes/macrophages is based on the TCRγ*δ*. Immunobiol. (2013) 218:960–8. 10.1016/j.imbio.2012.11.005 23312956

[19] Fuchs T Puellmann K Emmert A Fleig J Oniga S Laird R. The macrophage-TCRα*β* is a cholesterol-responsive combinatorial immune receptor and implicated in atherosclerosis. Biochem Biophys Res Commun. (2015) 456:59–65. 10.1016/j.bbrc.2014.11.034 25446098

[20] Oakley MS Chorazeczewski JK Aleshnick M Anantharaman V Majam V Chawla B. TCRβ-expressing macrophages induced by a pathogenic murine malaria correlate with parasite burden and enhanced phagocytic activity. PLoS ONE. (2018) 13:e0201043 10.1371/journal.pone.0201043 30044851 PMC6059462

[21] Fuchs T Hahn M Riabov V Yin S Kzhyshkowska J Busch S. A combinatorial α*β* T cell receptor expressed by macrophages in the tumor microenvironment. Immunobiology. (2017) 222:39–44. 10.1016/j.imbio.2015.09.022 26494401

[22] Kaminski WE Beham AW Puellmann K. Extralymphocytic flexible immune recognition: a new angle on inflammation and aging. Aging Dis. (2012) 3:404–13. 23185720 PMC3501395

[23] Kaminski WE Beham AW Kzhyshkowska J Gratchev A Puellmann K. On the horizon: Flexible immune recognition outside lymphocytes. Immunobiology. (2013) 218:418–26. 10.1016/j.imbio.2012.05.024 22749215

[24] Huang J Sun X Gong X He Z Chen L Qiu X. Rearrangement and expression of the immunoglobulin mu-chain gene in human myeloid cells. Cell Mol Immunol. (2014) 11:94–104. 10.1038/cmi.2013.45 24141767 PMC4002143

[25] Wang C Xia M Sun X He Z Hu F Chen L IGK with conserved IGKV/IGKJ repertoire is expressed in acute myeloid leukemia and promotes leukemic cell migration. Oncotarget. (2015) 6:39062–72. 10.18632/oncotarget.5393 26429876 PMC4770757

[26] Fuchs T Hahn M Ries L Giesler S Busch S Wang C. Expression of combinatorial immunoglobulins in macrophages in the tumor microenvironment. PLoS ONE. (2018) 13:e0204108. 10.1371/journal.pone.0204108 30240437 PMC6150476

[27] Pannetier C Cochet M Darche S Casrouge A Zoller M Kourilsky P. The sizes of the CDR3 hypervariable regions of the murine T-cell receptor beta chains vary as a function of the recombined germ-line segments. Proc Natl Acad Sci USA. (1993) 90:4319–23. 10.1073/pnas.90.9.4319 8483950 PMC46498

[28] Wang C Sanders CM Yang Q Schroeder HW Jr Wang E Babrzadeh F. High throughput sequencing reveals a complex pattern of dynamic interrelationships among human T cell subsets. ProcNatl Acad Sci USA. (2010) 107:1518–23. 10.1073/pnas.0913939107 20080641 PMC2824416

[29] Oehler L Majdic O Pickl WF Stöckl J Riedl E Drach J. Neutrophil granulocyte-committed cells can be driven to acquire dendritic cell characteristics. J Exp Med. (1998) 187:1019–28. 10.1084/jem.187.7.1019 9529318 PMC2212207

[30] Schatz DG Oettinger MA Baltimore D. The V(D)J recombination activating gene, RAG-1. Cell. (1989) 59:1035–48. 10.1016/0092-8674(89)90760-5 2598259

[31] Dreyfus DH. Immune system: success owed to a virus? Science. (2009) 325:392–3. 10.1126/science.325_392c 19628838

[32] Dreyfus DH. Paleo-immunology: evidence consistent with insertion of a primordial herpes virus-like element in the origins of acquired immunity. PLoS ONE. (2009) 4:e5778. 10.1371/journal.pone.0005778 19492059 PMC2686171

