# Supplementary material for: Immediate Neutrophil-Variable-T Cell Receptor Host Response in Bacterial Meningitis
Source: Front Neurol. 2019 Apr 2;10:307. doi: 10.3389/fneur.2019.00307 (PMC6454057; doi:10.3389/fneur.2019.00307)
Supplement: Supplementary file 1 [file Data_Sheet_1.pdf]

Supplementary Material

Supplementary Table S1

Patient characteristics, co-morbidity and baseline laboratory parameters of patients with acute bacterial and viral meningitis

Supplementary Table S2

High-throughput sequence reads of the TCRβ CDR3 variants from patient 1, 2 and 3

Supplementary Figures

S1: CSF-cytology from patients 1,2,3,6
S2: CSF-cytology from patient 12
S3: Isotype control staining
S4: CSF-cytology from patient 4 in acute and post-acute phase
S5: TCRβ immunofluorescence stainings
S6: Confocal immunofluorescence microscopy of patients 2 and 11
S7: Mass-spectrometry identification of TCRβ variants
S8: Detection of genomic V(D)J rearrangements
S9: IL-8 levels in CSF and PB
S10: Repertoire diversities in PB- and CSF-neutrophils
S11: Repertoire analyses by CDR3 spectratyping
S12: CDR3 Repertoire analyses on the sequence level
S13: NGS TCRβ transcriptome analyses
S14: Quantitative length variant analyses
S15: Vβ gene usage of neutrophils and lymphocytes from the CSF
S16: Relative abundance of TCRβ CDR3 transcript variants
S17: Repertoire changes after ex vivo exposure to bacterial pathogens
S18: Ex vivo targeting of baits to the TCRαβ enhances neutrophil phagocytosis
S19: Analyses of TCRαβ-directed bead uptake
S20: Enhanced TCRαβ-directed phagocytosis in neutrophils

Additional *unpublished* material for editors and referees: Expression of the neutrophil TCR in septic arthritis, infected catheter wound and acute pleuritis
