# Supplementary material for: Immediate Neutrophil-Variable-T Cell Receptor Host Response in Bacterial Meningitis
Source: Front Neurol. 2019 Apr 2;10:307. doi: 10.3389/fneur.2019.00307 (PMC6454057; doi:10.3389/fneur.2019.00307)
Supplement: Supplementary file 2 [file Data_Sheet_2.pdf]

Figure S1A

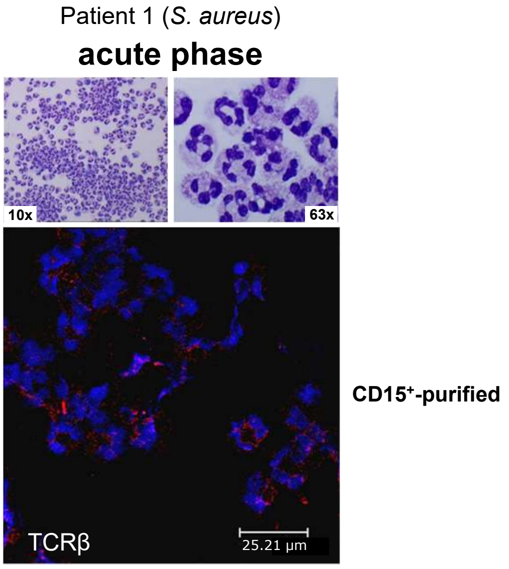

CSF-cytology image from patient 1 with acute *S. aureus* meningitis. The top row in the panel represents Giemsa stained CSF-cytospin preparations. The presence of bacteria (asterisk) is shown at a higher magnification (right). Confocal immunofluorescence microscopy (bottom) shows TCRβ expressing CD15^{+}-purified CSF neutrophils (red). Nuclei (blue) are counterstained with DRAQ5.

Figure S1B

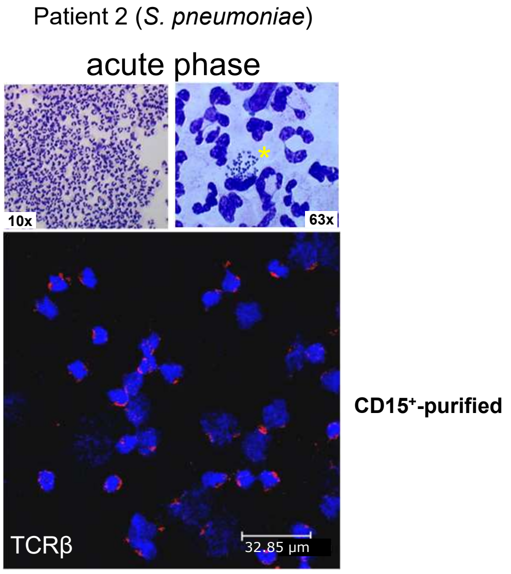

CSF-cytology image from patient 2 with acute S. pneumoniae meningitis. Details as in legend Fig. S1A

Figure S1C

Patient 3 (*E. coli*)

acute phase

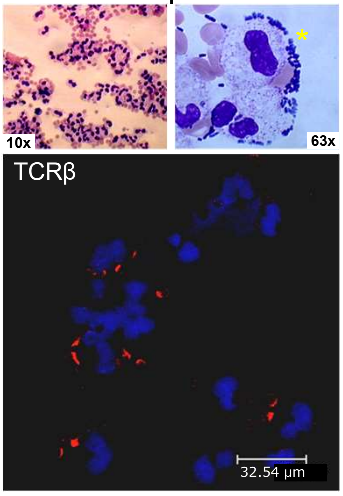

CD15^{+}-purified

CSF-cytology image from patient 3 with acute *E.coli* meningitis.
Details as in legend Fig. S1A

Figure S1D

Patient 6 (*E. coli*)

acute phase

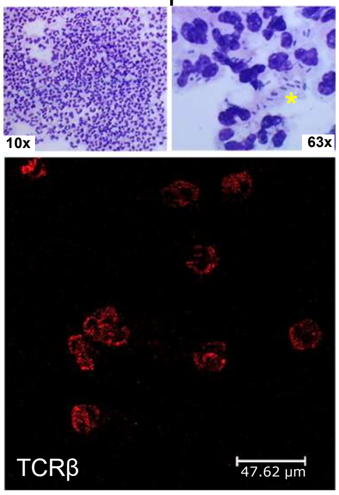

CD15^{+}-purified

CSF-cytology image from patient 6 with acute *E.coli* meningitis. Details as in legend Fig. S1A.
Confocal immunofluorescence microscopy (bottom). Nuclei unstained.

Figure S2

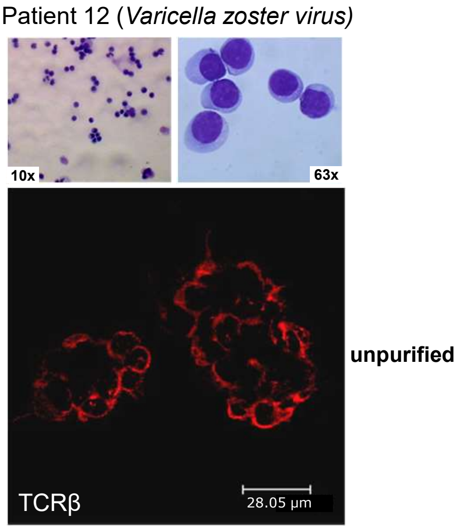

CSF-cytology from a patient with viral meningitis (*Varicella zoster* virus). Positive control for TCRβ immunocytochemistry. Shown are CSF cytology and TCRβ expression in a patient with acute viral meningitis caused by *varicella zoster* virus. Cytospin preparations and TCRβ immunostainings (red) were performed under identical conditions as for the patients with bacterial meningitis with the exception that cells were not MACS-purified. Note marked TCRαβ expression by CSF lymphocytes.

Figure S3

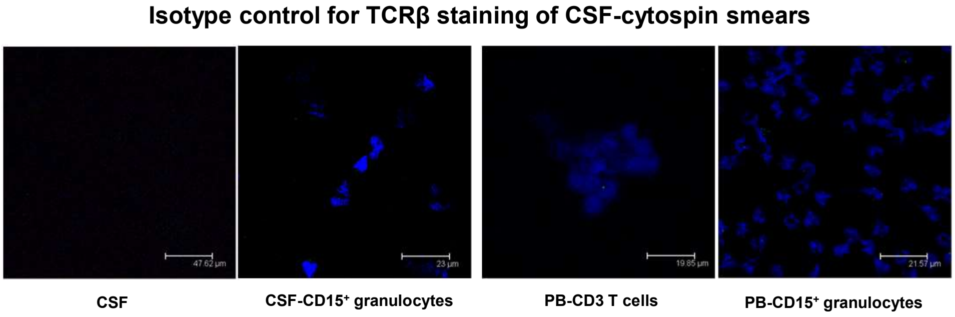

Isotype control stainings of CSF and peripheral blood. Isotype control staining of unpurified (CSF, left) and various MACS-purified leukocyte samples from CSF or peripheral blood (PB), respectively. Note that consistently no immunostaining was observed when isotype-matched control antibodies were used in place of the anti-TCRab (mouse IgG1) primary antibody. Nuclei (blue) are counterstained with DRAQ5 (except the left panel). The samples were collected from patients 1, 2, 6 and 7, respectively.

Figure S4

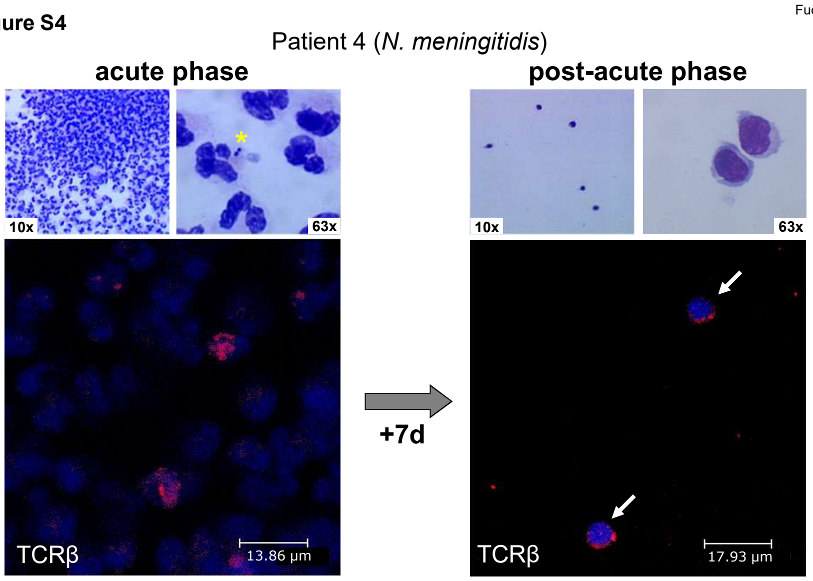

CSF-cytology from patient 4 with *N. meningitidis* meningitis: acute vs. post-acute phase. CSF cytology and TCRβ expression in the acute vs. post-acute phase of N. meningitidis meningitis. Shown are cytospin preparations and TCRβ immunostainings (red) from CSF collected 8 h after onset of symptoms (acute phase) and after 7 days of standard antibiotic therapy (post-acute phase). Note that the TCRβ positive and negative neutrophils are substituted by TCRβ expressing T cells (arrows). Bacteria are highlighted by a yellow asterisk.

Figure S5

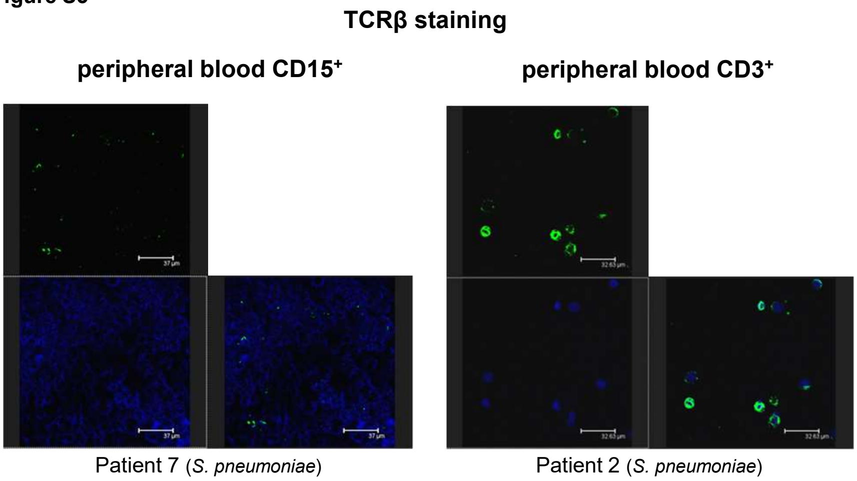

TCRβ immunofluorescence staining of peripheral blood neutrophils and lymphocytes in patients with acute *S. pneumoniae* meningitis. Confocal immunofluorescence microscopy demonstrating TCRβ positive (green) $CD15^+$-purified neutrophils (left) and $CD3^+$-purified T cells (right) in peripheral blood from two patients with acute bacterial meningits. Note that only a minor subpopulation of peripheral blood neutrophils express the TCRβ (left). In each panel the bottom right quadrant represents the merged image of the two left quadrants. $CD15^+$ neutrophils and $CD3^+$ T cells were purified by MACS before immunostaining. Nuclei are counterstained with DRAQ5.

Figure S6A

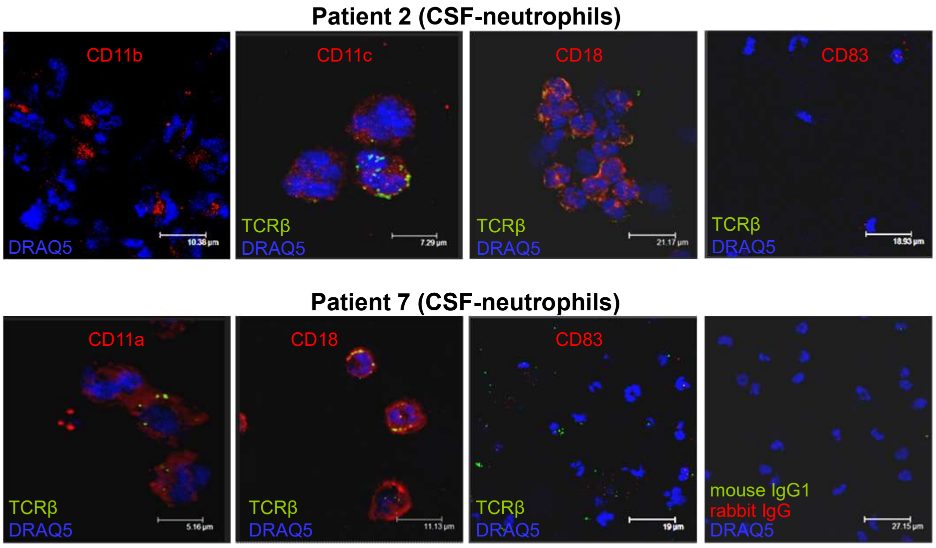

Confocal immunofluorescence microscopy shows consistent expression of canonical neutrophil markers (CD11a, CD11b, CD11c, CD18) in CSF neutrophils from two representative patients. The lineage markers CD11a, CD11b, CD11c, CD18 and CD83, respectively, are stained red, and the TCRβ is stained green. Nuclei (blue) are counterstained with DRAQ5. The bottom right panel shows representative isotype controls. Note the absence of the dendritic cell marker CD83 from CSF neutrophils.

Figure S6B

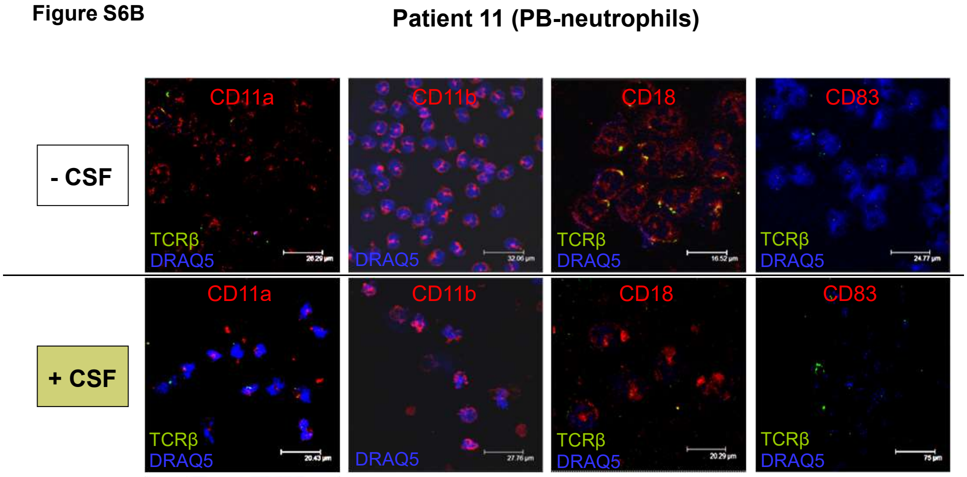

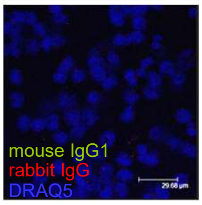

Septic CSF does not alter neutrophil-specific lineage marker expression in neutrophils. Freshly isolated $CD15^+$ PB neutrophils from a patient with *Enterococcus faecalis* meningitis (patient 11) were stained for the indicated neutrophil markers (red), the dendritic cell marker CD83 (red) and TCRβ (green) before (-CSF) and after incubation with the patient's own CSF (+CSF, 14 h). Note consistent expression of CD11a, CD11b and CD18, respectively, and the absence of CD83 from neutrophils irrespective of the presence of septic CSF. A representative isotype control is shown in the bottom left panel. Nuclei (blue), DRAQ5.

Figure S7A

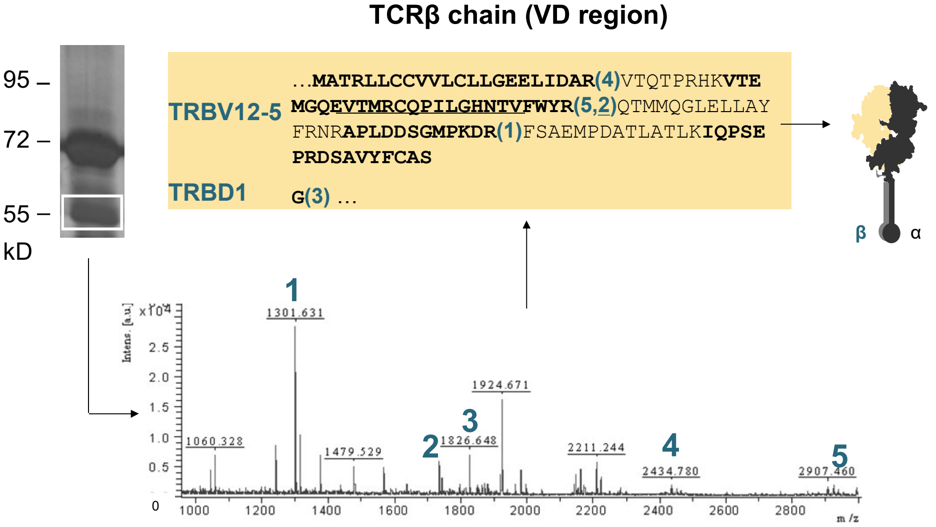

Mass-spectrometric identification of a rearranged Vβ-chain variant in CSF neutrophils from patient 3. Mass-spectrometric identification of a rearranged TCR Vβ-chain in CSF neutrophils from patient 3 (*E. coli* meningitis). Protein lysates from the patient's CSF CD15+-neutrophils were immunoprecipitated using an anti-TCRβ antibody and the predicted band (boxed) was analyzed by MALDI-TOF mass spectrometry. Peaks 1-5 represent TCR Vβ -specific peptide fragments. Amino acid sequence identities with known TCR Vβ -chains are bolded. They are consistent with a Vβ8-Dβ1 rearranged clonotype.

Fuchs et al.

Figure S7B

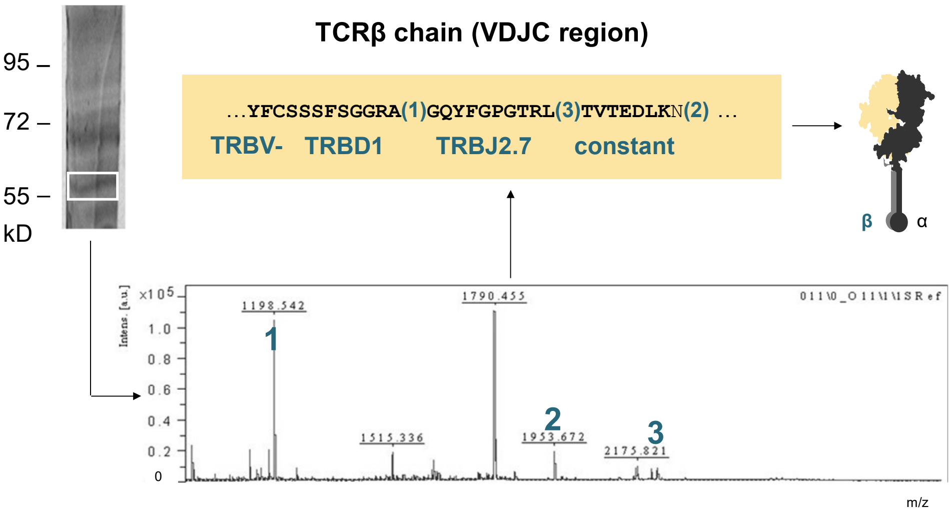

Mass-spectrometric identification of a rearranged Vβ-chain variant in CSF neutrophils from patient 7. Mass-spectrometric identification of a rearranged TCR Vβ -chain in CSF neutrophils from patient 7 (*S. pneumoniae* meningitis).
For details see legend Fig. S7A. The identified TCR variant peptides (1-3) are consistent with a TRBV-TRBD1-TRBJ2.7 clonotype.

Figure S8

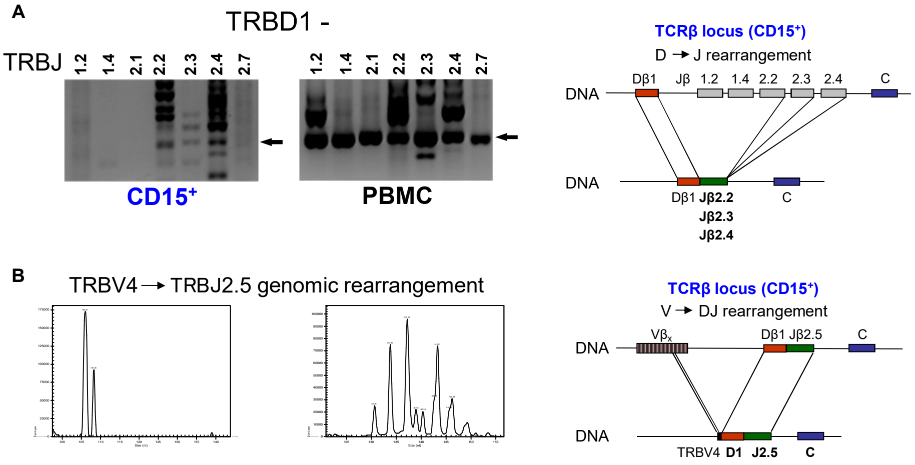

Detection of D → J and V → DJ rearrangement in the TCRβ gene locus of human CD15⁺ neutrophils. (A) Arrows denote the presence of TRBD1 → TRBJx rearrangements in human peripheral blood CD15⁺ neutrophils and mononuclear cells (PBMC, positive controls) which were confirmed by sequencing. TRBV→ TRBD/TRBJ rearrangement is representatively shown for TRBV4 and TRBJ2.5 using complementary determining region 3 (CDR3) length spectratyping (B). Genomic organization of the identified rearrangements are schematically drawn (right). Rearrangement of the TCRβ gene locus we tested using a PCR assay based on the protocols established by van Dongen et al. (Leukemia 17: 2257-2317, 2003) in combination with sequencing of specific amplification products.

Figure S9

Fuchs et al.

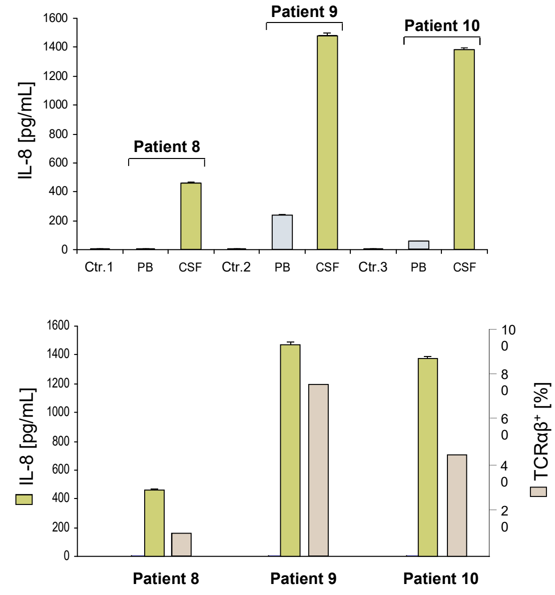

PB and CSF IL-8 levels in three randomly selected patients
Ctr.1-3, healthy age-matched PB controls for patients 8, 9, and 10. IL-8 concentrations and the corresponding percentages of TCRαβ+ neutrophils in the CSF are shown in the bottom panel.

Figures S10

Following page:
Repertoire diversities of the TCR variable β-chains that are expressed by PB- and CSF neutrophils from patients 1, 2 and 3, respectively, during acute-phase meningitis.
RT-PCR expression profiling was performed for all 25 known human TCR Vβ chains (Vβ1-25). The scattergrams (top) document that the $CD15^+$ neutrophils are free of T lymphoid cells (CD2). For each patient, expressed TCR Vβ chains are represented by filled boxes and their respective designations are indicated. Empty boxes represent nonexpressed Vβ chains. Note the marked increase in expressed TCR Vβ chain genes in $CD15^+$ CSF neutrophils relative to $CD15^+$ PB neutrophils. The detailed repertoires assessed by length variant analysis of the antigen-binding CDR3β region ("CDR3β spectratyping") in each of the expressed Vβ chains are shown.

Fuchs et al.

Figure S10

Fuchs et al.

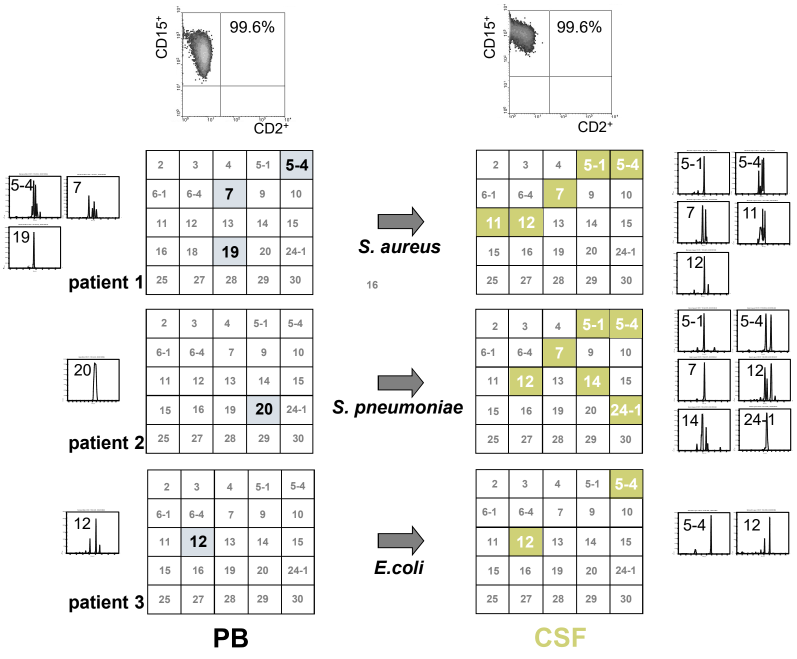

Figure S11

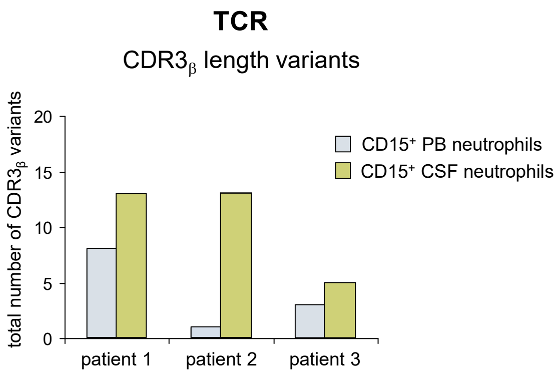

Repertoire analyses by CDR3β spectratyping in all three patients reveal that the total numbers of the expressed TCRβ CDR3 length variants are consistently higher in CD15[+] CSF neutrophils than in CD15[+] PB neutrophils indicative of repertoire broadening at the site of inflammation.

Figure S12

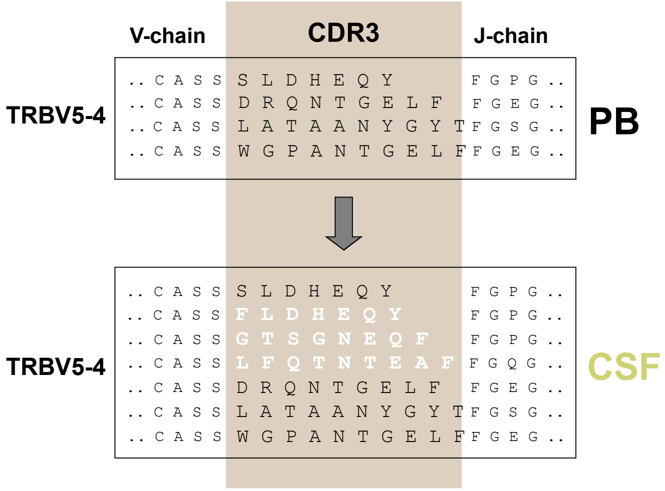

CDR3β repertoire broadening in CSF neutrophils (bottom) relative to PB neutrophils (top) shown on the detailed clonotype level. Amino acid (aa) sequences of expressed TRBV5-4 chain variants are representatively shown for patient 1. Identified CDR3β segments (shaded area) span 7-10 aa displaying a similar length variation as those expressed by T cells. The sequences of the flanking V- and J-chain segments are indicated. Note expression of three additional TRBV5-4 clonotypes in CSF neutrophils (white) that were not detected in PB neutrophils. The TRBV5-4 variants were identified by cloning and sequencing.

Figures S13

Following pages:
NGS TCRβ transcriptome analyses reveal induction of the TCR in CSF neutrophils during the acute phase of bacterial meningitis.

(top) Repertoire diversity tree plots visualize the relative abundance of the TCRβ CDR3 transcript variants that are expressed by CD15^{+} neutrophils in CSF and in peripheral blood of patient 2 (Fig S13a) and patient 3 (Fig. S13b). Each spot represents a rearranged TCRβ transcript that encodes a unique CDR3β sequence. It is defined by a unique color and its area is proportional to the relative transcript frequency. The position of each spot within the plot area is defined according to its Vβ usage (x-axis: Vβ1→Vβi) and Jβ usage (y-axis: Jβ1→Jβi). Each plot has a distinct color code. Total numbers of identified nonredundant ("unique") TCRβ CDR3 sequence variants are indicated for each diversity tree plot. CDR3, complementarity determining region 3.

(bottom) Detailed list of the 10 most frequently expressed TCRβ CDR3 variants in each neutrophil population. Transcript copy numbers (f) are indicated. TCRβ variants that are shared between PB and CSF neutrophils are connected by arrows. TCRβ variants that show an increase in CSF neutrophils are highlighted in blue.

Figure S13A

Patient 2

CD15^{+} PB neutrophils | CD15^{+} CSF neutrophils

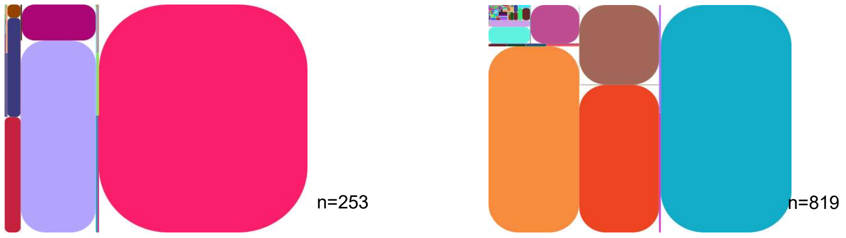

CDR3 (peptide)	Copy	CDR3 (peptide)	Copy
ASSSLDHEQY	42,561	ASSSLDHEQY	1,029,960
ASSLATAANYGYT	12,891	ASRTSTGSGNTIY	585,326
ASSGTSGNEQF	2,380	ASSLATAANYGYT	407,648
ASSFQAGGSYEQY	1,651	ASSGTSGNEQF	219,488
ASSWGPANTGELF	1,147	ASSPSGAAYEQY	64,584
ASRTSTGSGNTIY	175	ASSWGPANTGELF	23,909
ASSSLDYEQY	166	ASSDRQNTGELF	9,389
ASSSLDHGQY	160	ASSSLGHEQY	3,627
ASSDRQNTGELF	153	ASRSLDHEQY	3,354
ASGSLDHEQY	127	ASSSLDHGQY	3,202

Figure S13B

Patient 3

CD15$^+$ PB neutrophils

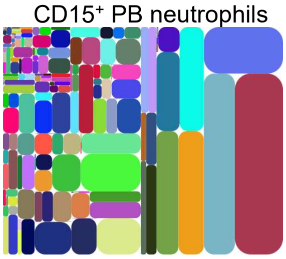

n=2,852

CD15$^+$ CSF neutrophils

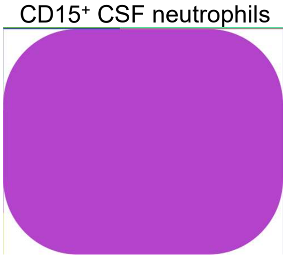

n=101

CDR3 (peptide)	Copy
ASSSSTEAF	850,020
ASSLGVEAF	546,926
ASSLYGVTEAF	359,135
ASSLGPEAF	306,055
ASSSGIEAF	263,159
ASSSGSLAF	244,781
SARGRGTDTQY	221,923
ASSWGTEAF	178,666
ASRASGTDTQY	156,514
SARVSSELDGYT	117,123

CDR3 (peptide)	Copy
ASSSLDHEQY	33,647
ASSSLDHGQY	93
ASSPLDHEQY	88
ASSSPDHEQY	66
ASSSLGHEQY	64
ASSLDGSEQY	15
AIVIRQSHNEQF	6
ASSLATAANYGYT	4
ASRTSTGSGNTIY	2
ASSLWGPYNEQF	2

Figure S14

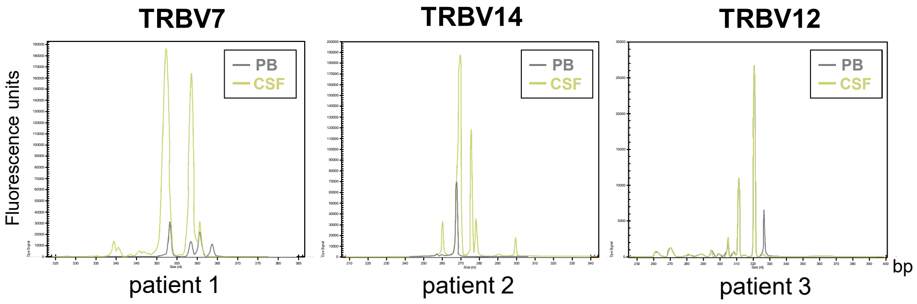

Quantitative length variant analysis of the antigen-binding CDR3β region in patients 1, 2 and 3 shows a dramatic increase in TCR Vβ repertoire gene expression levels in CSF neutrophils compared to PB neutrophils as exemplified for three TCR variable chains (TRBV7, TRBV14 and TRBV12). Peak heights are indicated as fluorescence units.

Figures S15

Following pages:
Vβ gene usage of CD15^{+} neutrophils and CD3^{+} lymphocytes, respectively, in peripheral blood and CSF of patient 1 (A) and patient 2 (B,C). The 2D-plots demonstrate the relative usage of the Vβ genes for each cell compartment. The results are shown as regular and normalized distributions. The normalized distribution counts the value (for V Gene usage) of each distinct CDR3 as one, no matter how many of the particular CDR3s are observed. In short, each CDR3-VDJ combination is treated as a quantity of 1 regardless of read count, and then analyzed for V usage. This allows for a view of the repertoire removing the skewing which may occur due to one or just a few highly dominant clones. The regular distribution is based on the number directly observed from the read count data. Note the markedly restricted Vβ gene usage in CD15^{+} cells relative to CD3^{+} cells. X-axis: Vβ gene; y-axis: percentage of used Vβ genes.

Figures S15A

Patient 1- normalized distribution

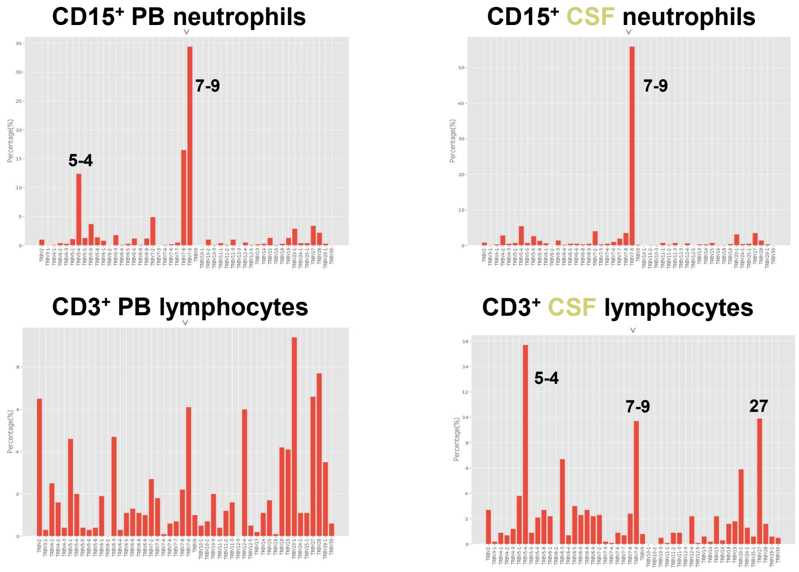

Figure S15B

Patient 2- regular distribution

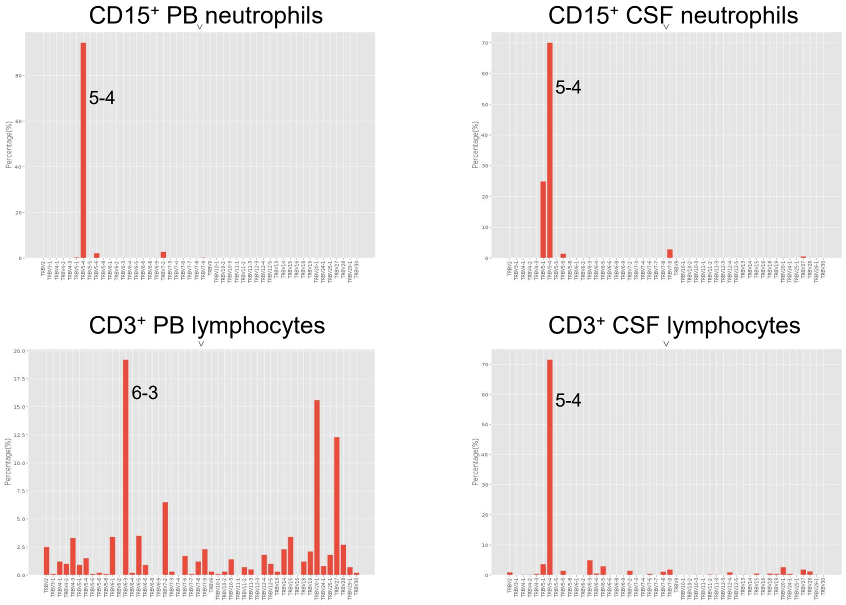

Figure S15C

Patient 2- normalized distribution

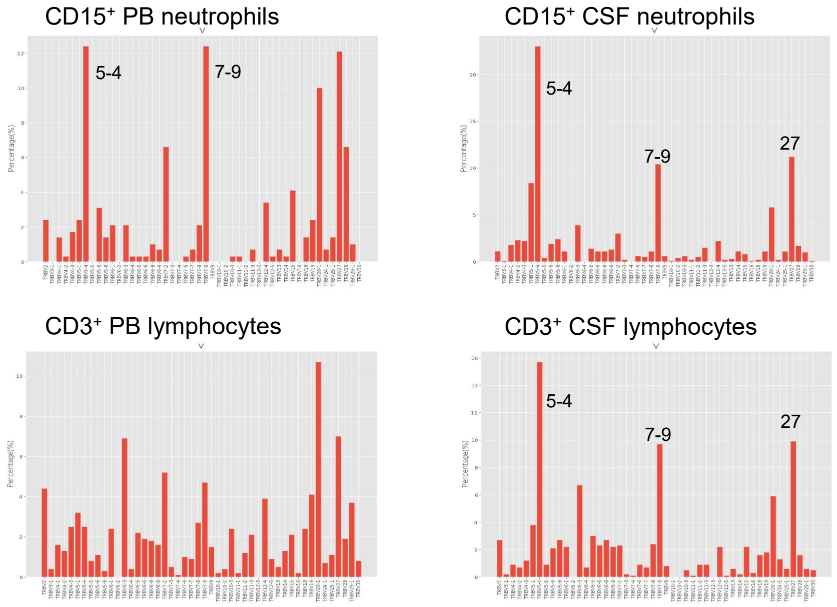

Figures S16

Following pages:
(top) Relative abundance of TCRβ CDR3 transcript variants expressed by CD15^{+} neutrophils and CD3^{+} lymphocytes in CSF (patient 1). Total numbers of identified nonredundant TCRβ CDR3 sequence variants are indicated for each leukocyte subpopulation.
(bottom) Comparison of the 10 most frequently expressed TCRβ CDR3 variants reveals that neutrophils and lymphocytes in CSF share common repertoires (arrows), but also express a similar proportion of distinct TCRβ variants. Numbers designate transcript copy numbers. Note that two of the shared TCRβ CR3 sequence variants exhibit higher expression rates in CSF neutrophils than in CSF lymphocytes (blue).

Figure S16

Patient 1

CD3^{+} CSF lymphocytes — CD15^{+} CSF neutrophils

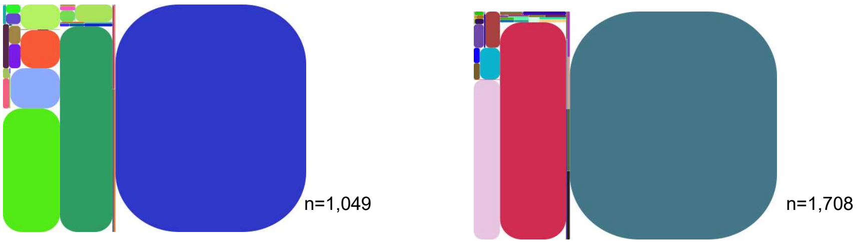

CDR3 (peptide)	Copy		CDR3 (peptide)	Copy
ASSLDGSEQY	4,452,372	→	ASSLDGSEQY	12,792,731
AIVIRQSHNEQF	1,107,779	→	AIVIRQSHNEQF	3,888,386
ASSLATAANYGYT	722,003	→	ASSLATAANYGYT	1,132,717
ASSSLDHEQY	204,174	→	ASSSLDHEQY	171,815
ASTLDEMNTEAF	154,743		ASSDRQNTGELF	143,776
SARTSNEQF	99,726		ASSLGGGGQETQY	63,656
ASSRVTGGVDEKLF	66,140		ASSPSGAAYEQY	51,421
ASSDRQNTGELF	28,832		ASSLDGSGQY	46,497
ASSPRRDEQY	26,884		ASSRVTGGVDEKLF	39,807
ASSPDGSEQY	24,988		ASSLGGSEQY	35,796

Figures S17

(Following pages):

***Ex vivo* exposure to bacterial pathogens that cause meningitis triggers dynamic TCR Vβ repertoire changes**. Figure S9 shows the TCR Vβ repertoires that are expressed by purified peripheral blood CD15^{+} neutrophils from two healthy subjects (2,3) after 9 h incubation with *N. meningitidis* (orange), *S. pneumoniae* (green) and in the absence of a bacterial pathogen, respectively (Ø, control, black). The detailed neutrophil-TCR Vβ repertoires are shown for each subject in the right panels. The purity of the CD15+ neutrophils is shown in the scattergrams (left). Note that *N. meningitidis* and *S. pneumoniae* induce distinct neutrophil-TCR Vβ chain usage (center) and CDR3β repertoires (right) in each subject.

Figure S17A

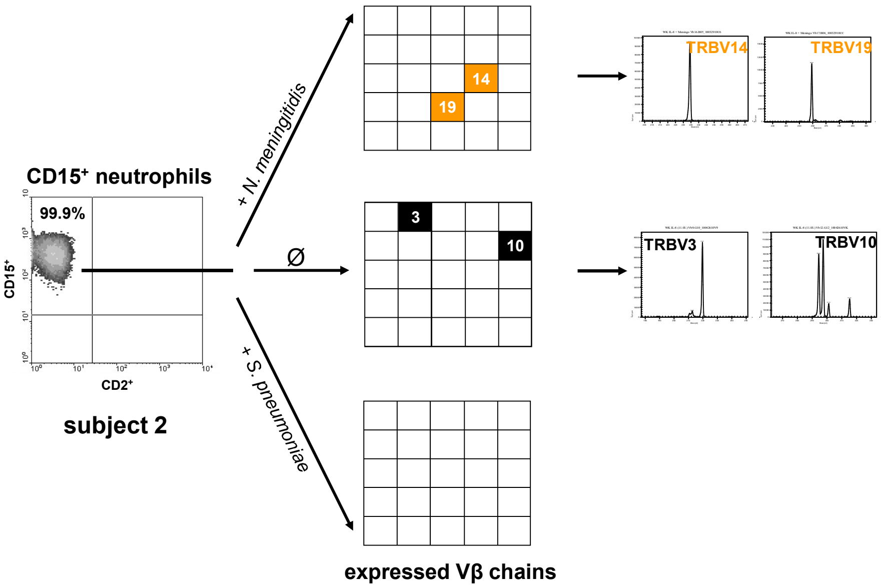

Figure S17B

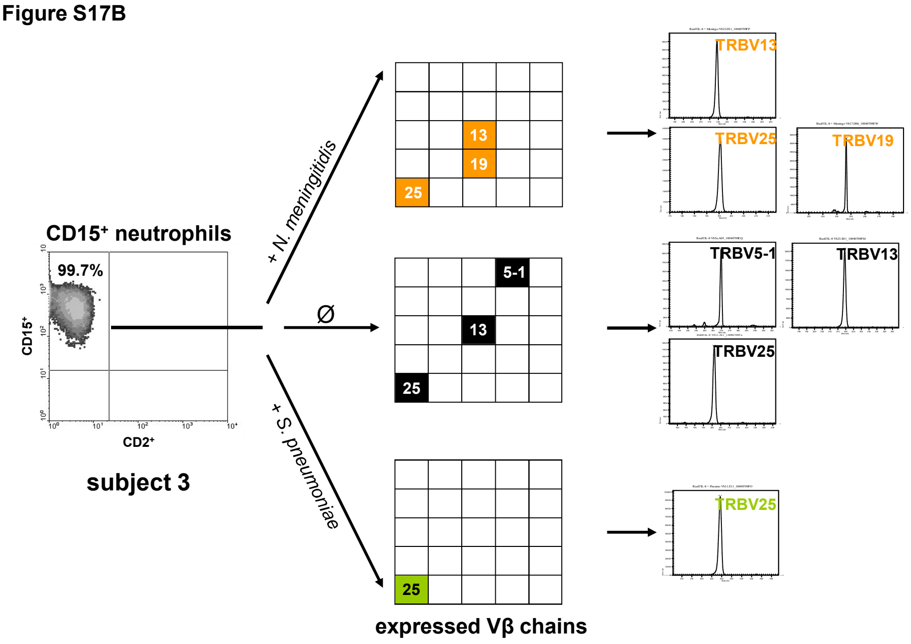

Fuchs et al.

Fuchs et al.

Figure S18

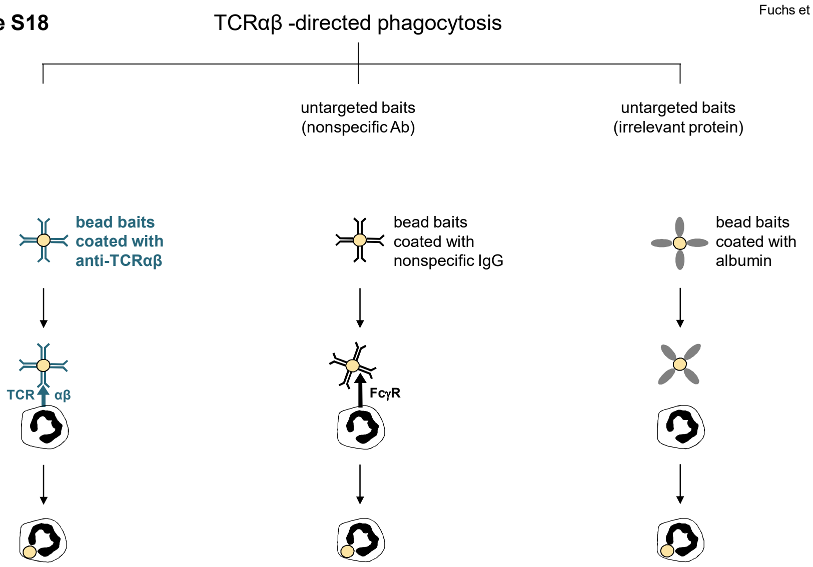

***Ex vivo* targeting of baits to the TCRαβ enhances neutrophil phagocytosis.** Schematic representation of phagocytosis assay used for *ex vivo* targeting of baits to the neutrophil TCRαβ. Neutrophils were challenged with polystyrene bead baits (Ø 4.5 µm) coated with anti-TCRαβ antibodies and uptake of beads was recorded. Beads coated with nonspecific IgG isotype antibodies, potentially binding to the Fcγ receptor (FcγR), or albumin (irrelevant protein) served as controls (untargeted baits).

Figure S19A

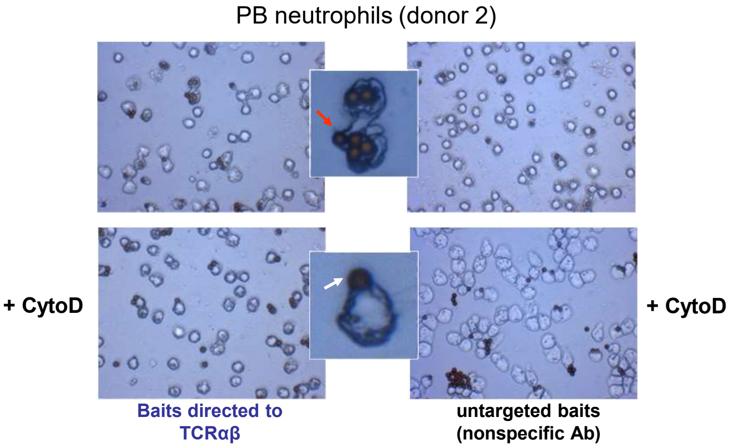

TCRαβ-directed bead uptake in intact neutrophils and during cytochalasin D-induced blockade of phagocytosis.
(A) Representative unstained cytospin preparations (20x) of purified CD15+ PB neutrophils from a normal individual (donor 2) that were co-incubated with bead baits targeted to the TCRαβ or untargeted beads (nonspecific IgG) for 3 hours. Cytochalasin D (5 µg/ml) treated neutrophils are shown in the bottom panel (+ CytoD). Right panel: untargeted control beads (nonspecific IgG). The red arrow highlights internalized beads at a higher magnification, the white arrow an external bead during inhibition of phagocytosis.

Figure S19B

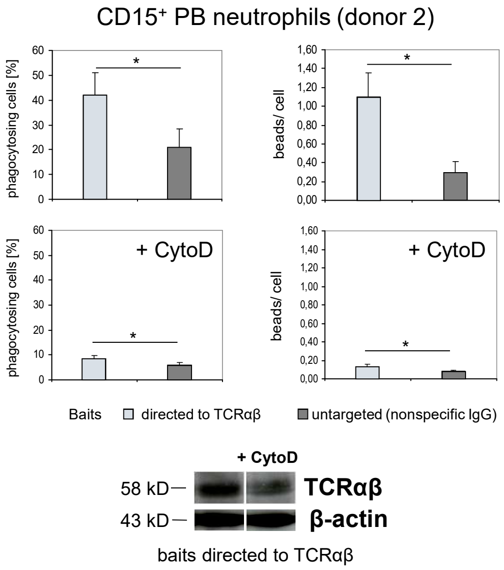

(B) Quantitative analysis of bead phagocytosis (percentage of phagocytosing cells, left; beads/cell, right) as shown in A. Note that neutrophil phagocytosis is enhanced when beads are directed to the TCRαβ and the pro-phagocytic effect is abrogated when phagocytosis is suppressed (+ CytoD). * p<0.01 (student's pair test). The immunoblot in the bottom panel demonstrates the presence of the TCRαβ in the neutrophils to which bead phagocytosis was directed. A loading control is shown (β-actin).

Figure S20

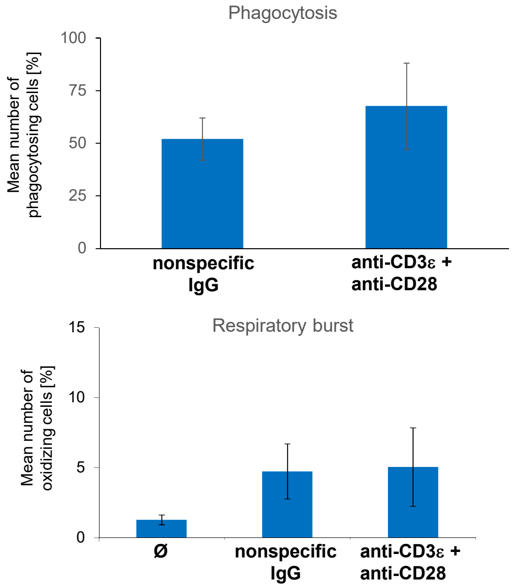

Enhanced TCRαβ-directed phagocytosis in neutrophils. Quantitative analysis of phagocytosis and respiratory burst (percentage of phagocytosing cells,top; oxidizing cells, bottom) assessed by flow cytometry. Note that neutrophil phagocytosis is enhanced when cells are stimulated with anti-CD3ε/anti-CD28 antibodies whereas the number of oxidizing cells is not affected. Mean number of phagocytosing/oxidizing cells from five (top) and three (bottom) independent healthy donors are shown, respectively.
