# Supplementary material for: Immediate Neutrophil-Variable-T Cell Receptor Host Response in Bacterial Meningitis
Source: Front Neurol. 2019 Apr 2;10:307. doi: 10.3389/fneur.2019.00307 (PMC6454057; doi:10.3389/fneur.2019.00307)
Supplement: Supplementary file 3 [file Data_Sheet_3.PDF]

Table S1 Patient characteristics, co-morbidity and baseline laboratory parameters of patients with acute bacterial (1-11) and viral meningitis (12)

Patient No. [1]	Sex	Age (y)	Pathogen	Community acquired (+/-)	Co-morbidity	CSF WBC (cells/µl)	CSF Lactate (mmol/l)	CSF Glucose (mg/dl)	peripheral blood WBC (x 10^3/µl)
1	m	75	*S. aureus*	–	sepsis<br>NIDDM	2455	13.7	75	12.4
2	m	60	*S. pneumoniae*	–	subarachnoid hemorrhage	8193	8.9	8	5.8
3	f	57	*E. coli*	–	subarachnoid hemorrhage<br>hemicraniectomy<br>agranulocytosis	791	13.9	1	6.4
4	f	63	*N. meningitidis*	+	–	8852	10.9	21	19.1
5	f	74	*S. pneumoniae*	+	osteoporosis<br>chronic renal disease<br>monoclonal gammopathy	545	20.0	<5	4.2
6	m	59	*E. coli*	–	stroke, hemicraniectomy<br>CAD	9919	17.9	<3	11.9
7	f	70	*S. pneumoniae*	+	stroke	9531	17.9	<5	18.7
8	m	52	*S. capitis*	–	discectomy	13000	6.5	31	16.3
9	m	30	*S. aureus*	–	thoracic epidural abscess	300	3.3	52	6.3
10	f	72	*S. salivarius*	–	lumbar ischalgia	15418	8.3	26	23.6
11 [2]	f	42	*Enterococcus faecalis*	–	subarachnoid hemorrhage	3815	10.1	1	10.2
12 [3]	m	24	*Varicella zoster virus*	+	–	291	2.8	42	5.0

[1] None of the patients had a history of acquired or congenital immunodeficiency disorders
[2] additional patient included for neutrophil activation experiments (Supplementary Fig S6B)
[3] patient with acute viral meningits (control)

NIDDM, non-insulin-dependent diabetes mellitus
CAD, coronary artery disease
