# Supplementary material for: Immediate Neutrophil-Variable-T Cell Receptor Host Response in Bacterial Meningitis
Source: Front Neurol. 2019 Apr 2;10:307. doi: 10.3389/fneur.2019.00307 (PMC6454057; doi:10.3389/fneur.2019.00307)
Supplement: Supplementary file 4 [file Data_Sheet_4.PDF]

Table S2 High-throughput sequence reads of the TCRβ CDR3 variants from patient 1, 2 and 3

Patient No.	$CD15^+$ isolated from	amount of RNA for reverse transcription [ng]	Number of effective reads [1] [n]	total $CDR3_β$ variants (productive) [n]	unique $CDR3_β$ variants (productive) [2] [n]
**1**	CSF	300	18733636	18726994	1708
	PB	400	1324252	1323473	575
**2**	CSF	400	2388173	2387809	819
	PB	400	62075	62046	253
**3**	CSF	300	34086	34082	101
	PB	300	6237201	6213639	2852

Fuchs et al.

[1] An effective read is a read that can be mapped to both V and J germline segments
[2] A unique CDR3 sequence is defined as a nonredundant fragment of amino acids which is derived from a stop-codon-free reading frame containing both translated conserved V and J motifs
$CD15^+$, neutrophils; CSF, cerebrospinal fluid; PB, peripheral blood; CDR3, complementarity determining region
